# Cerebral malaria: of mice and men

**DOI:** 10.1093/trstmh/traf126

**Published:** 2025-11-14

**Authors:** Chamarika J Weerasekera, Nicholas J White

**Affiliations:** Department of Parasitology, Faculty of Medical Sciences, University of Sri Jayewardenepura, Gangodawila, Nugegoda, Sri Lanka; Mahidol–Oxford Tropical Medicine Research Unit, Faculty of Tropical Medicine, Mahidol University, Bangkok 10400, Thailand; Mahidol–Oxford Tropical Medicine Research Unit, Faculty of Tropical Medicine, Mahidol University, Bangkok 10400, Thailand; Centre for Tropical Medicine and Global Health, Nuffield Department of Medicine, University of Oxford, Oxford OX3 7LF, UK

**Keywords:** adjuvant treatment, cerebral malaria, mouse model, severe malaria

## Abstract

**Background:**

Cerebral malaria is a major cause of death in endemic areas. An animal model of cerebral malaria has been studied widely in which C57BL/6 mice are infected with the *Plasmodium berghei* ANKA strain. The histopathology and the response to interventions of human cerebral malaria and the murine model are very different. In 2012, a consensus guideline was published recommending that in order to represent better the clinical setting, interventions in the murine model should be tested together with antimalarial drug treatment and after development of the cerebral syndrome.

**Methods:**

A systematic review of publications on human and murine cerebral malaria since 2010 was conducted.

**Results:**

Clinical research on human cerebral malaria has declined and still no adjuvant intervention has proved effective. Meanwhile, since 2010, 149 interventions (118 adjuvants) have been evaluated in the mouse model, of which 142 (95%) were reportedly successful. Only 26% of interventions were evaluated after the development of the murine cerebral syndrome and 65% of the adjuvants were tested without a concomitant antimalarial.

**Conclusion:**

The predictive value of the murine model in identifying adjuvant therapeutic interventions in human cerebral malaria is very poor.

## Introduction

Cerebral malaria, the most characteristic syndrome of severe falciparum malaria,^1^ is a diffuse, symmetrical, potentially lethal febrile encephalopathy that may occur at any age.^2^ The treated mortality of cerebral malaria is usually 8–15%, depending on the extent of other vital organ dysfunction.^1,3^ Although most patients who survive cerebral malaria recover without evident deficit, a significant proportion of patients, particularly children, do have residual neurological sequelae and there are significant late complications that may arise, such as epilepsy. Cerebral malaria is therefore both a major cause of preventable death in tropical countries and an important cause of chronic morbidity. In lower transmission settings the clinical syndrome of coma together with *Plasmodium falciparum* parasitaemia is distinctive and specific, but in higher transmission settings where the disease is confined to younger children, the diagnosis is more difficult. Cerebral malaria is misdiagnosed in 20–30% of febrile parasitaemic comatose children.^4,5^ These misdiagnosed children have incidental parasitaemia and another cause for their coma. It is currently estimated that some 250 million people are infected with malaria each year, of whom approximately 600 000 die. The majority of these preventable deaths are in children in sub-Saharan Africa.^6^

The pathogenesis of cerebral malaria has intrigued scientists and clinicians for more than a century. It is generally accepted that the seminal pathological observations and deductions of Marchiafava and Bignami^7^ are correct; that is, that cerebral malaria results from the extensive sequestration of erythrocytes containing mature *P. falciparum* parasites in the cerebral microvasculature. Mechanical obstruction of cerebral microvascular flow is undoubtedly a critical pathophysiological component, but how exactly this causes coma and death remains unclear.^1,8^ Although sequestration is observed in some other primate malarias (*Plasmodium coatneyi, Plasmodium fragile*), extensive cerebral sequestration of parasitized erythrocytes is unique to human cerebral malaria.

While clinical (bedside) investigation of malaria in endemic areas has declined, laboratory research has expanded in temperate regions. To facilitate laboratory research, an animal model of cerebral malaria has been proposed and studied widely in which C57BL/6 mice are infected with the *Plasmodium berghei* ANKA strain. In this murine model, neurological signs precede death, yet the pathology and the responses to interventions are very different to that of human cerebral malaria. Instead of the cardinal pathological signature of parasitised erythrocyte sequestration, there is extensive leukocyte accumulation in the cerebral microvasculature in murine models.

A major purpose of animal models is to inform clinical management and treatment. An extensive list (nearly 200 in total since the murine model was described first) of potential ‘adjuvant’ therapies have been evaluated in the mouse ‘cerebral malaria’ model. Nearly all have been successful (although many of the test interventions were given before development of the syndrome and they were often given without concomitant antimalarial drugs). In stark contrast, none of the adjuvants tested in humans have proved effective, and some have been harmful. This disparity has naturally provoked discussion and debate as to the clinical relevance of the experimental murine model. A consensus review^14^ convened in 2010 and published 13 years ago recommended that adjuvants tested in the murine model should be given alongside specific antimalarial treatment to reproduce the likely clinical context (i.e. the experimental intervention evaluated in the laboratory mice should be administered after rather than before the cerebral syndrome developed). The aim of this article was to review the recent literature on interventional studies in murine and human cerebral malaria, to determine the impact of the consensus recommendations on therapeutic studies in animal malarias^14^ and to evaluate critically the clinical implications.

## Methods

We reviewed the recent bibliography of the interventional studies of murine cerebral malaria and their implications for causal inference in pathophysiological processes. A literature search of studies published in English or French between 1 January 2010 and 31 December 2024 listed in the National Library of Medicine (PubMed) was carried out using the following MeSH search terms: cerebral malaria; subheadings, therapy, drug therapy, diet therapy, pathophysiology, pathology.^9^ The studies were checked manually for cerebral malaria studies on humans and mice. Articles not directly pertaining to severe malaria, reviews, editorials, letters, notes and case reports were excluded.

## Results

**Figure 1.**
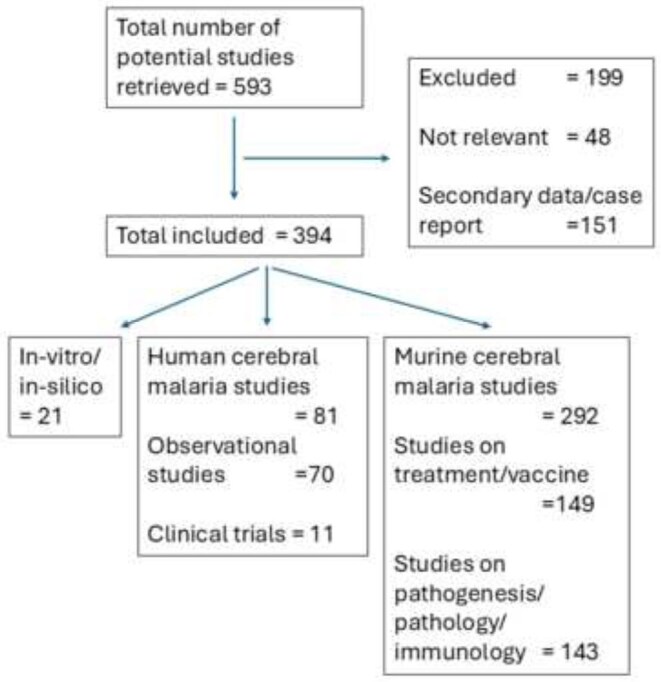
Comparing human and murine cerebral malaria studies since 2010.

Of the 593 studies retrieved, 394 comprised original research pertaining to ‘cerebral malaria’. Of these, 292 (78%) were on murine cerebral malaria, 81 were on human cerebral malaria and 21 did not describe in vivo studies (Figure 1). Of the murine cerebral malaria studies, 149 evaluated treatment strategies (known antimalarials or novel treatments, either as monotherapies or as adjunctive therapy) or were vaccine studies. Meanwhile 11 clinical trials in human cerebral malaria were reported in the past 15 y. Apart from imaging and histopathology studies, there were 25 additional investigations that studied pathophysiological mechanisms and assessed biomarkers.

**Figure 2.**
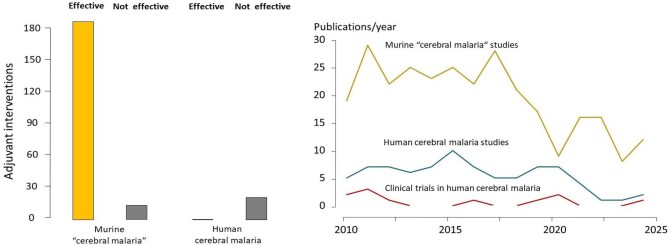
(Left) The reported efficacy of all interventions in human and murine ‘cerebral malaria’ in published studies. (Right) Published studies on cerebral malaria from 2010 to 2024.

Since 2019 there has been an overall decrease by about half in both murine and human cerebral malaria studies (Figure 2). The top four contributing countries for murine cerebral malaria studies were the USA, China, Brazil and India, accounting to more than half of the studies. Fewer than 10 murine studies originated from sub-Saharan Africa. The differences between human cerebral malaria and murine cerebral malaria and the differences and similarities in neuroimaging and ocular findings and in the neuropathology findings between post-mortem studies of adults in Southeast Asia and children studied in Malawi have all been reviewed extensively.^1^ Here we compare and contrast therapeutic responses to interventions in human and murine cerebral malaria.

## Assessment of interventions

Since 2010, 120 different interventions have been evaluated in 149 studies on the murine cerebral malaria model (see Supplementary Table S1). Nearly all of these have been successful (Figure 2). Of these, 118 involved adjuvants and the others were antimalarial drugs. Only seven (vitamin E [S53], endothelin 1 [S122], vitamin A+DTP [S231], lambda-carrageenan [S94], hydrogen sulphide [S88], L-arginine [S113] and high dietary folate [S118]) were either neutral or worsened the murine disease. However, hydrogen sulphide gas and L-arginine were both shown to be effective in other murine cerebral malaria studies [S129, S138, S154] (Supplementary Table S1). Thus 94.4% of the adjuvant interventions (95% confidence interval 90.3% to 98.4%) were beneficial in the murine model.

Between 2010 and 2024, 11 clinical trials in human cerebral malaria were conducted (see Supplementary Table S2). Ten clinical studies evaluated seven adjunct therapies (aggressive antipyretic therapy [S238], delayed iron therapy [S235–S237], vitamin A [S231], mannitol [S224, S228], early enteral feeding [S230], pentoxifylline [S207, S211, S212, S217] and inhaled nitric oxide [S31, S58]). Two interventions (mannitol and early enteral feeding) were harmful [S228, S230]. The trial on aggressive antipyretic therapy (an open comparison of paracetamol plus ibuprofen versus paracetamol alone) reported a reduced incidence of multiple or prolonged seizures but raised concerns over renal impairment from ibuprofen (S238). The potential benefit of albumin therapy suggested by a preliminary study was not confirmed in a much larger clinical trial.^15,16^ None of the interventions was clearly beneficial (Figure 2). Co-infection with *Schistosoma mansoni*, chikungunya virus and filaria have also been shown to be beneficial in mice, but there are no convincing data in humans. Thus, despite the very high success rate of interventions in experimental cerebral malaria, these findings have not been translated into benefit in human cerebral malaria.

The mortality of untreated cerebral malaria approaches 100%. This is reduced by a factor of 5–6 by quinine treatment. The quinine-treated mortality is reduced by a further third by parenteral artesunate.^1,8^ In 2010, the consensus meeting agreed that interventions in animal models should be tested together with antimalarial drugs to reflect the context of potential use.^14^ Unfortunately, this sensible advice has often not been followed. Since 2010, 65% of the adjuvant intervention studies in mice (75 of 118; 4 were excluded as they were vaccination studies) did not test the adjunct with an antimalarial and 74% of the murine cerebral malaria studies (110 of 149) examined the benefits of treatment administered before the development of cerebral signs.

## Discussion

While global malaria mortality is estimated to have increased since 2015, clinical bedside research on severe malaria has decreased (Figure 2). A search performed on two clinical trial registration sites (ClinicalTrials.gov and the International Standard Randomised Controlled Trial Number [ISRCTN]) identified no ongoing phase 3 trials and only two phase 1 or phase 2 trials (ISCRTN76942974 and ISRCTN79071535). Studies on murine cerebral malaria still comprise the majority of experimental studies on severe malaria. Animal models should reproduce the clinical and pathological processes that occur in human disease. The extraordinary success (95%) of most interventions in this particular murine model, a combined list that now approaches 200 potential adjuvants since the first murine study was reported in 1987, stands in stark contrast to the absence of benefit demonstrated for any adjuvant intervention in humans. This remarkable discrepancy, and the sheer diversity of chemicals that work in the mouse, must raise serious concerns over the therapeutic relevance of the murine cerebral malaria model and the studies performed on it.

Experimental studies on mice conducted in a good laboratory animal facility are easier to perform than clinical studies on human cerebral malaria in resource-limited malaria-endemic areas. Despite the disparities, protagonists of the murine model point to some qualitative similarities in cerebral pathology, while conceding that there are also major histopathological differences. Nevertheless, it is often argued that some aspects of the murine model are useful in guiding therapeutic strategies. But which ones? Today the only interventions shown to be beneficial in the mouse model that are being taken forward in human clinical trials are inhaled nitric oxide (S233, S234), erythropoietin (NCT00697164) and 6-diazo-5-oxo-L-norleucine (a potent inhibitor of glutamine metabolism and T cell proliferation; NCT05478720).^17^ It is notable that dexamethasone and anti-tumour necrosis factor antibody were beneficial in the murine model and harmful in randomized controlled trials in humans, while prostacyclin (iloprost), pentoxifylline and oral activated charcoal were beneficial in the murine model but not in humans (S209, S217, S226, S227).

With limited funding for malaria research and a severe decrease in clinical investigations, it is important to question the relevance of experimental systems such as the murine cerebral malaria model. In contrast, the other main manifestations of severe falciparum malaria (anaemia, metabolic acidosis, increased pulmonary capillary permeability and acute kidney injury) may have closer parallels in animal models and thus merit further experimental animal study, although the criteria for validation are unclear. However, the hypothesis that the murine model of cerebral malaria can be used to identify treatments of human cerebral malaria cannot be sustained. Use of the term cerebral malaria for the murine model should be discontinued.

## Supplementary Material

traf126_Supplemental_Files

## Data Availability

All data are in the references in the supplementary lists.
