# Supplementary material for: Cerebral malaria: of mice and men
Source: Trans R Soc Trop Med Hyg. 2025 Nov 14;120(3):254–7. doi: 10.1093/trstmh/traf126 (PMC13017480; doi:10.1093/trstmh/traf126)
Supplement: traf126_Supplemental_Files [file traf126_supplemental_files.zip › Supplementary files.docx]

**Supplementary Tables and references**

**Table S1.**

**Full referenced list of interventions evaluated in the murine model of cerebral malaria from 1987 to 2024.**

|  | **Intervention** | **Effect** | **Parasite** | **Mice** | **Authors** |
| --- | --- | --- | --- | --- | --- |
| [S1] | Anti-TNF antibody | Reduced CM Improved survival | *Plasmodium berghei* ANKA | CBA/Ca | Grau *et al.,* 1987 |
| [S2] | Cyclosporine | Reduced CM Improved survival | *Plasmodium berghei* ANKA | CBA/Ca | Grau *et al.,* 1988 |
| [S3] | anti-rGM-CSF and anti-rIL-3 antibodies | Reduced CM | *Plasmodium berghei* ANKA | CBA | Grau *et al.,* 1988 |
| [S4] | Antioxidants | Reduced CM | *Plasmodium berghei* ANKA | A/J and CBA/H | Thurmwood *et al.,* 1989 |
| [S5] | Anti gamma interferon antibody | Reduced CM Improved survival | *Plasmodium berghei* ANKA | CBA/Ca | Grau *et al.,* 1989 |
| [S6] | IL-1 | Reduced CM Improved survival | *Plasmodium berghei* K 173 | C57Bl/6J | Curfs *et al.,* 1990 |
| [S7] | Anti LFA1 (CD11a) antibody | Reduced CM Improved survival | *Plasmodium berghei* ANKA | CBA/Ca | Grau *et al.,* 1991 |
| [S8] | Pentoxifylline | Reduced CM Improved survival | *Plasmodium berghei* ANKA | CBA/Ca | Kremsner *et al.,* 1991 |
| [S9] | Iloprost | Reduced CM Improved survival | *Plasmodium berghei* ANKA | CBA/Ca | Sliwa *et al.,* 1991 |
| [S10] | Fish Oil | Reduced CM Improved survival | *Plasmodium berghei* ANKA | C57B1/6 | Blok *et al.,* 1992 |
| [S11] | Indomethacin | No effect | *Plasmodium berghei* ANKA | C57B1/6 | Blok *et al.,* 1992 |
| [S12] | Starvation | Prevention of CM | *Plasmodium berghei* ANKA | CBA/T6 | Hunt *et al.,* 1993 |
| [S13] | Murine AIDS | Prevention of CM | *Plasmodium berghei* ANKA | C57/BL/6 | Eckwalanga *et al.,* 1995 |
| [S14] | Fish oil | Prevention of CM | *Plasmodium berghei* ANKA | CBA/CaJ | Levander *et al.,* 1995 |
| [S15] | Dexamethasone | Reduced CM Improved survival | *Plasmodium berghei* ANKA | CBA/T6 and DBA/2J | Neill & Hunt 1995 |
| [S16] | Fatty acids | Reduced CM | *Plasmodium berghei* ANKA | C57/BL/6 | Moumaris *et al.,* 1995 |
| [S17] | Coinfection with *B.pahangi* | Reduced CM Improved survival | *Plasmodium berghei* | CBA/J | Yan *et al.,* 1997 |
| [S18] | CD4 or CD8 depletion | Reduced CM | *Plasmodium berghei* K 173 | C57B1/6J and C57B1/10 | Hermsen *et al.,* 1997 |
| [S19] | Recombinant IL-10 | Reduced CM | *Plasmodium berghei* ANKA | CBA/J | Kossodo *et al.,* 1997 |
| [S20] | Splenectomy | Reduced CM Improved survival | *Plasmodium berghei* K 173 | C57BL/6J | Hermsen *et al.,* 1998 |
| [S21] | Thiolated recombinant TNF (rhTNF alpha-AT) | Reduced CM Improved survival | *Plasmodium berghei* K173 | C57BL/6J | Postma *et al.,* 1999 |
| [S22] | Liposomal recombinant TNF | Reduced CM Improved survival | *Plasmodium berghei* K173 | C57BL/6J | Postma *et al.,* 1999 |
| [S23] | Dichloroacetate | Improved survival | *Plasmodium berghei* ANKA | CBA/T6 | Rae *et al.,* 2000 |
| [S24] | Immunisation with synthetic GPI | Prevents CM and death | *Plasmodium berghei* ANKA | C57BL6 | Schofield *et al.,* 2002 |
| [S25] | Anti CD 41 | Reduced CM Improved survival | *Plasmodium berghei* ANKA | C57BL/6 | Sun *et al.,* 2003 |
| [S26] | Anti CD 61 | Reduced CM Improved survival | *Plasmodium berghei* ANKA | C57BL/6 | Sun *et al.,* 2003 |
| [S27] | Celecoxib | Earlier onset of CM | *Plasmodium berghei* ANKA | CBA or C57BL6 | Ball *et al.,* 2004 |
| [S28] | kynurenine-3-hydroxylase inhibitor | Reduced CM Improved survival | *Plasmodium berghei* ANKA | C57BL/6J | Clark *et al.,* 2005 |
| [S29] | Thalidomide | Improved survival | *Plasmodium berghei* ANKA | CBA | Muniz-Junqueira *et al.,* 2005 |
| [S30] | Erythropoietin | Improved survival | *Plasmodium berghei* ANKA | CBA/J | Kaiser *et al.,* 2006 |
| [S31] | Nitric oxide | Reduced CM Improved survival | *Plasmodium berghei* ANKA | C57BL/6 | Gramaglia *et al.,* 2006 |
| [S32] | anti-BTLA mAb | Reduced CM | *Plasmodium berghei* ANKA | C57BL/6 | Lepenies *et al.,* 2007 |
| [S33] | Carbon monoxide | Reduced CM Improved survival | *Plasmodium berghei* ANKA | C57BL/6 | Pamplona *et al.,* 2007 |
| [S34] | Recombinant human IFN-alpha | Reduced CM Improved survival | *Plasmodium berghei* ANKA | C57BL/6 | Vigario *et al.,* 2007 |
| [S35] | anti CD25 antibody | Reduced CM Improved survival | *Plasmodium berghei* ANKA | CBA and C57BL/6 | Amante *et al.,* 2007 |
| [S36] | CD8 T cell depletion | Prevention or attenuation of CM | *Plasmodium berghei* ANKA | CBA and C57BL/6 | Randall *et al.,* 2008 |
| [S37] | Guanylhydrazone CNI-1493 | Reduced CM Improved survival | *Plasmodium berghei* ANKA | C57BL/6 | Specht *et al.,* 2008 |
| [S38] | Antibody blockade of C5a or C5a receptor (C5aR) | Reduced CM Improved survival | *Plasmodium berghei* ANKA | C57BL/6 | Patel *et al.,* 2008 |
| [S39] | Pantethine | Reduced CM Improved survival | *Plasmodium berghei* ANKA | CBA/J | Penet *et al.,* 2008 |
| [S40] | Simvastatin | No effect | *Plasmodium berghei* ANKA | C57BL/6 | Kobbe *et al.,* 2008 |
| [S41] | Aspirin | Reduced CM Improved survival | *Plasmodium berghei* ANKA | C57BL/6 | Srivastava *et al.,* 2008 |
| [S42] | Clopidogrel | Reduced CM Improved survival | *Plasmodium berghei* ANKA | C57BL/6 | Srivastava *et al.,* 2008 |
| [S43] | Anti-apoptotic strategies | No effect | *Plasmodium berghei* ANKA | C57BL/6 | Helmers *et al.,* 2008 |
| [S44] | Obesity | Prevented CM Improved survival | *Plasmodium berghei* ANKA | C57BL/6 | Robert *et al.,* 2008 |
| [S45] | Hyperbaric oxygen | Prevented CM Improved survival | *Plasmodium berghei* ANKA | C57BL/6 | Blanco *et al.,* 2008 |
| [S46] | Glatiramer | Reduced CM Improved survival | *Plasmodium berghei* ANKA | C57BL/6 | Lackmer *et al.,* 2009 |
| [S47] | IP 10 blockade | Reduced CM Improved survival | *Plasmodium berghei* ANKA | C57BL/6 | Nie *et al.,* 2009 |
| [S48] | Rosiglitazone | Reduced CM Improved survival | *Plasmodium berghei* ANKA | C57BL/6 | Serghides *et al.*, 2009 |
| [S49]* | Artemisone together with chloroquine | Complete cure | *Plasmodium berghei* ANKA | C57BL/6 | Waknine-Grinberg *et al.,* 2010 |
| [S50] | Rodent filarial infection by *Litomosoides sigmodontis* | Reduced inflammation | *Plasmodium berghei ANKA* | C57BL/6 | Specht *et al.,*2010 |
| [S51] | Fasudil | Prevented CM | *Plasmodium berghei* ANKA | *ICR* | Waknine-Grinberg *et al.,*2010 |
| [S52] | *S.mansoni* infection | Reduced CM Improved survival | *Plasmodium berghei* ANKA | C57BL/6 | Waknine-Grinberg *et al.,*2010 |
| [S53] | Vitamin E | No improvement in symptoms, parasitaemia and survival | *Plasmodium berghei* ANKA | C57BL/6 | Herbas *et al.,* 2010 |
| [S54] | Nimodipine | Increased survival | *Plasmodium berghei* ANKA | C57BL/6 | Cabrales *et al.,* 2010 |
| [S55] | Vitamin A + DTP | Increased parasitaemia Increased CM | *Plasmodium berghei* ANKA | C57BL/6 | Hein-Kristensen *et al.,*2010 |
| [S56] | Oral activated charcoal | Prevented CM | *Plasmodium berghei* ANKA | C57BL/6 | de Souza *et al.,* 2010 |
| [S57] | IL-2/anti-IL-2 complexes | Prevented CM | *Plasmodium berghei* ANKA | C57BL/6 | Haque *et al.,*2010 |
| [S58] | Inhaled nitric oxide | Improved CM  Reduced parasitaemia Improved survival | *Plasmodium berghei* ANKA | C57BL/6 | Serghides *et al.,*2011 |
| [S59] | lentiviral vector-mediated overexpression of hepcidin (pLenti-Hep-EGFP) | Reduced parasitaemia Improved survival | *Plasmodium berghei* ANKA | ICR and Kunming strain | Wang *et al.,*2011 |
| [S60] | Sphingosine 1- pathway modulators: FTY720, LX2931 | Improved survival | *Plasmodium berghei* ANKA | C57BL/6 | Finney *et al.,*2011 |
| [S61] | NO donor (dipropylenetriamine NONOate [DPTA-NO]) | Prevented CM | *Plasmodium berghei* ANKA | C57BL/6 | Cabrales *et al.,* 2011 |
| [S62] | Amodiaquine-derived NO-donor, | Prolonged survival | *Plasmodium berghei* ANKA | C57BL/6 | Bertinaria *et al.,*2011 |
| [S63] | TLR7 and TLR9 antagonist, E6446 | Prevented complications/ inflammation | *Plasmodium berghei* ANKA | C57BL/6 | Franklin *et al.,*2011 |
| [S64] | Beta interferon | Reduced inflammation Improved CM | *Plasmodium berghei* ANKA | C57BL/6 | Morrell *et al.,* 2011 |
| [S65]* | Artemether | Cured CM | *Plasmodium berghei* ANKA | C57BL/6 | Clemmer *et al.,* 2011 |
| [S66] | Attenuated Plasmodium *berghei* parasites | Reduced parasitaemia Prevented CM | *Plasmodium berghei* ANKA | C57BL/6 | Gerald *et al.,* 2011 |
| [S67] | FMS-like tyrosine kinase 3 (FLT3) ligand | Prevented CM | *Plasmodium berghei* ANKA | C57BL/6 | Tamura *et al.,* 2011 |
| [S68] | S-nitrosoglutathione, an NO donor | Prevented CM | *Plasmodium berghei* ANKA | C57BL/6 | Zanini *et al.,* 2011 |
| [S69] | Lovastatin | Prevented inflammation and CM | *Plasmodium berghei* ANKA | C57BL/6 | Reis *et al.,*2012 |
| [S70] | Lithium | Reduced neurocognitive impairment | *Plasmodium berghei* ANKA | C57BL/6 | Dai *et al.,* 2012 |
| [S71] | ETA receptor antagonist HJP-272 | Improved survival | *Plasmodium berghei* ANKA | C57BL/6 | Dai *et al.,* 2012 |
| [S72] | Erythropoietin | Reduced severity of CM Improved survival | *Plasmodium berghei* ANKA | C57BL/6 | Hempel *et al.,* 2012 |
| [S73]* | Chloroquine | Prevented CM | *Plasmodium berghei* ANKA | C57BL/6 | Zhu *et al.,* 2012 |
| [S74] | S-nitrosoglutathione (GSNO) | Reduced parasitaemia Prevented CM | *Plasmodium berghei* ANKA | C57BL/6 | Zanini *et al.,* 2012 |
| [S75] | sildenafil plus DPTA-NO | Reduced ECM | *Plasmodium berghei* ANKA | C57BL/6 | Martins *et al.,* 2012 |
| [S76] | Atorvastatin | Improved survival Reduced parasitaemia Prevented CM | *Plasmodium berghei* ANKA | CBA/J | Souraud *et al.,* 2012 |
| [S77] | Carbon monoxide-releasing molecule (CO-RM; ALF492) | Prevented CM | *Plasmodium berghei* ANKA | C57BL/6 | Pena *et al.,* 2012 |
| [S78]* | Artemiside | Prolonged survival Reduced parasitaemia | *Plasmodium berghei* ANKA | C57BL/6 | Guo *et al.,* 2012 |
| [S79]* | Artesunate | Reduced inflammation Reduced cognitive impairment Improved survival | *Plasmodium berghei* ANKA | C57BL/6 | Miranda *et al.,* 2013 |
| [S80] | Glucocorticosteroids in nano-sterically stabilized liposomes | Complete cure Reduced inflammation Reduced sequelae | *Plasmodium berghei* ANKA | C57BL/6 and ICR | Waknine-Grinberg *et al.,* 2013 |
| [S81] | Atorvastatin | Reduced CM Improved survival Reduced parasitaemia | *Plasmodium berghei* ANKA | C57BL/6 | Dormoi *et al.,* 2013 |
| [S82] | *Azadirachta indica* ethanolic extract | Prevented complications | *Plasmodium berghei* ANKA | Swiss albino mice | Bedri *et al.,* 2013 |
| [S83] | Transdermal glyceryl trinitrate | Increased survival Reduced complications | *Plasmodium berghei* ANKA | C57BL/6 | Orjuela-Sánchez *et al.,* 2013 |
| [S84] | Atorvastatin | Reduced CM improved survival | *Plasmodium berghei* ANKA | C57BL/6 | Wilson *et al.,* 2013 |
| [S85] | Nimodipine | Increased survival Improved CM | *Plasmodium berghei* ANKA | C57BL/6 | Martins *et al.,*2013 |
| [S86]* | Methylene blue | Improved CM Improved survival Reduced parasitaemia | *Plasmodium berghei* ANKA | C57BL/6 | Dormoi *et al.,* 2013 |
| [S87] | Methylene blue and atorvastatin | Improved survival | *Plasmodium berghei* ANKA | C57BL/6 | Dormoiivar *et al.,*2013 |
| [S88] | Hydrogen sulfide gas | Not effective | *Plasmodium berghei* ANKA | C57BL/6 | DellaValle *et al.,*2013 |
| [S89] | Reversible JNK pathway inhibitor SP600125 | Improved survival Reduced neuronal cell death | *Plasmodium berghei* ANKA | C57BL/6 | Anand *et al.,*2013 |
| [S90] | rh-Erythropoietin | Improved CM | *Plasmodium berghei* ANKA | C57BL/6 | Karlsson *et al.,* 2013 |
| [S91] | Erythropoietin | Improved survival | *Plasmodium berghei* ANKA | C57BL/6 | Wei *et al.,*2013 |
| [S92] | Lipoxin A₄ and 15-epi-lipoxin A₄ | Prolonged survival | *Plasmodium berghei* ANKA | C57BL/6 | Shryock *et al.,* 2013 |
| [S93]* | Intranasal artesunate | Improved survival Reduced parasitaemia | *Plasmodium berghei* ANKA | CBA/J mice | Marijon *et al.,* 2014 |
| [S94] | Lambda-carrageenan | Increased severity  Increased mortality | *Plasmodium berghei* ANKA | BALB/c mice | Recuenco *et al.,* 2014 |
| [S95] | Liposome-encapsulated betamethasone hemisuccinate (nSSL-BMS) | Prevented CM Increased survival | *Plasmodium berghei* ANKA | C57BL/6 | Guoalry *et al.,* 2014 |
| [S96] | Vitamin D | Improved CM | *Plasmodium berghei* ANKA | C57BL/6 | He *et al.,* 2014 |
| [S97]* | Artemisone in combination with conventional antimalarial drugs | Prevented CM Reduced parasitaemia | *Plasmodium berghei* ANKA | C57BL/6 | Guiguemde *et al.,* 2014 |
| [S98] | PPARγ agonists | Reduced neurocognitive impairment | *Plasmodium berghei* ANKA | C57BL/6 | Serghides *et al.,* 2014 |
| [S99] | Tempol | Partial protection against CM | *Plasmodium berghei* ANKA | C57BL/6 | Francischetti *et al.,* 2014 |
| [S100] | Neuregulin-1 | Increased parasites. Improved survival | *Plasmodium berghei* ANKA | C57BL/6 | Solomon *et al.,* 2014 |
| [S101] | Citicoline | Reduced parasitaemia Increased survival prevent CM | *Plasmodium berghei* ANKA | CBA | El-Assaad *et al.,* 2014 |
| [S102] | 6-diazo-5-oxo-L-norleucine (DON) | Improved CM | *Plasmodium berghei* ANKA | C57BL/6 | Gordon *et al.,* 2015 |
| [S103] | Vascular endothelial growth factor (VEGF) and lovastatin | Prevented CM | *Plasmodium berghei* ANKA | C57BL/6 | Canavese *et al.,*2015 |
| [S104] | VEGF and LPS | Prevented CM Improved survival | *Plasmodium berghei* ANKA | C57BL/6, Balb/c | Canavese *et al.,* 2015 |
| [S105] | Angiotensin -2 | Prevented CM Increased survival | *Plasmodium berghei* ANKA | C57BL/6 | Gallego-Delgado *et al.,* 2015 |
| [S106] | NO-Donor | Increased survival | *Plasmodium berghei* ANKA | C57BL/6 | Bertinaria *et al.,* 2015 |
| [S107] | Proteasome inhibitors - bortezomib | No improvement in survival | *Plasmodium berghei* ANKA | C57BL/6 | Howland *et al.,* 2015 |
| [S108] | Formulated diet to control iron status | Improved survival | *Plasmodium berghei* ANKA | H67D | Leitner *et al.,* 2015 |
| [S109] | Curcumin | Reduced inflammation | *Plasmodium berghei* ANKA | C57BL/6 | Dende *et al.,* 2015 |
| [S110] | Rapamycin inhibitor | Prevented CM | *Plasmodium berghei* ANKA | C57BL/6 | Gordon *et al.,* 2015 |
| [S111] | RRx-001  Anticancer drug | Improved inflammation/CM | *Plasmodium berghei* ANKA | C57BL/6 | Yalcin *et al.,* 2015 |
| [S112] | Mesenchymal stromal cell therapy | Increased survival Reduced parasitaemia | *Plasmodium berghei* ANKA | C57BL/6 | Souza *et al.,* 2015 |
| [S113] | L-arginine | Reduced survival  Increased inflammation | *Plasmodium berghei* ANKA | C57BL/6 | Xu *et al.,* 2015 |
| [S114] | Iron dextran | Prevented CM | *Plasmodium berghei* ANKA | C57BL/6 | Van *et al.,* 2015 |
| [S115] | Dietary restriction | Reduced parasite accumulation in brain | *Plasmodium berghei* ANKA | C57BL/6 | Mejia *et al.,* 2015 |
| [S116] | Cannabidiol | Increased survival  Rescue of cognitive function | *Plasmodium berghei* ANKA | C57BL/6 | Campos *et al.,* 2015 |
| [S117] | *Agaricus blazei* extract | Reduced CM Reduced parasitaemia Increased survival Reduced symptoms | *Plasmodium berghei* ANKA | C57BL/6 | Val *et al.,* 2015 |
| [S118] | High dietary folate | Higher parasitaemia  Reduced survival | *Plasmodium berghei* ANKA | C57BL/6 | Meadows *et al.,* 2015 |
| [S119]* | Nanostructured lipid carriers of artemether-lumefantrine | Improved CM | *Plasmodium berghei* ANKA | C57BL/6 | Prabhu *et al.,* 2016 |
| [S120] | Intramuscular vitamin D | Cured CM | *Plasmodium berghei* ANKA | C57BL/6 | Dwivedi *et al.,* 2016 |
| [S121] | Cysteamine | Reduced parasitaemia Prevented CM | *Plasmodium berghei* ANKA | A/J mice | Moradin *et al.,* 2016 |
| [S122] | Endothelin-1 | No effect on parasitaemia Increased severity of CM | *Plasmodium berghei* ANKA | C57BL/6 | Martins *et al.,* 2016 |
| [S123] | Synthetic oleanane triterpenoids | Improved survival Improved CM | *Plasmodium berghei* ANKA | C57BL/6 | Crowley *et al.,* 2017 |
| [S124] | Rapamycin | Improved survival Improved CM | *Plasmodium berghei* ANKA | C57BL/6 | Mejia *et al.,* 2017 |
| [S125] | Nanocurcumin | Inhibited sequestration | *Plasmodium berghei* ANKA | C57BL/6 | Dende *et al.,* 2017 |
| [S126] | Protein Tyrosine Phosphatase Inhibition | Prevented CM | *Plasmodium berghei* ANKA | C57BL/6 | van den Ham *et al.,* 2017 |
| [S127] | MEK1/2 inhibitor | Reduced parasitaemia Improved CM | *Plasmodium berghei* ANKA | C57BL/6 | Wu *et al.,* 2017 |
| [S128]* | Trioxaquine | Reduced parasitaemia Improved survival | *Plasmodium berghei* ANKA | C57BL/6 | Odhiambo *et al.,* 2017 |
| [S129] | Hydrogen sulfide | Improved CM | *Plasmodium berghei* ANKA | C57BL/6 | Jiang *et al.,* 2017 |
| [S130] | Erythropoietin | Improved CM Improved survival | *Plasmodium berghei* ANKA | C57BL/6 | Du *et al.,* 2017 |
| [S131] | *Zizyphus spina* extract (ZLE) | Improved CM | *Plasmodium berghei* ANKA | C57BL/6 | Mubarakid *et al.,* 2017 |
| [S132]* | Controlled release artemisone | Prevented CM | *Plasmodium berghei* ANKA | C57BL/6 | Golenser *et al.,* 2017 |
| [S133] | Trappin-2/elafin | Reduced parasitaemia | *Plasmodium berghei* ANKA | C57BL/6 | Roussilhon *et al.,* 2017 |
| [S134]* | Minocycline | Improved survival Prevented sequelae Reduced parasitaemia | *Plasmodium berghei* ANKA | C57BL/6 | Apoorv *et al.,*  2017 |
| [S135] | Tacrolimus | Prevented CM | *Plasmodium berghei* ANKA | C57BL/6 | Bao *et al.,* 2017 |
| [S136] | Glutamate receptor antagonist MK801 | Prevented sequelae | *Plasmodium berghei* ANKA | C57BL/6 | de Miranda *et al.,* 2017 |
| [S137] | Glutaminase inhibitor JHU-083 | Improved CM Improved survival | *Plasmodium berghei* ANKA | C57BL/6 | Riggle *et al.,* 2018 |
| [S138] | L-arginine | Improved survival | *Plasmodium berghei* ANKA | C57BL/6 | Ong *et al.,* 2018 |
| [S139] | Vitamin D | Improved CM Improved survival | *Plasmodium berghei* ANKA | C57BL/6 | Wu *et al.,* 2018 |
| [S140]* | Artesunate combination therapy | Reduced parasitaemia Improved CM Increased survival | *Plasmodium berghei* ANKA | C57BL/6 | Jiang *et al.,* 2018 |
| [S141] | α-Tocopheryl succinate | Reduced parasitaemia Increased survival | *P. yoelii* 17XL and *P. berghei* ANKA | C57BL/6 mice | Kume *et al.,* 2018 |
| [S142] | IL-33 | Improved CM Improved survival | *Plasmodium berghei* ANKA | C57BL/6 mice | Strangward *et al.,* 2018 |
| [S143] | Neuregulin-1 | Improved survival | *Plasmodium berghei* ANKA | C57BL/6 mice | Liu *et al.,* 2018 |
| [S144] | Interleukin-15 Complex Treatment | Improved survival | *Plasmodium berghei* ANKA | C57BL/6 mice | Burrack *et al.,* 2018 |
| [S145] | Rocaglate | Reduced parasitaemia Improve CM | *Plasmodium berghei* ANKA | C57BL/6 mice | Langlais *et al.,* 2018 |
| [S146] | Single intravenous or subcutaneous whole-parasite immunization | Delayed parasitaemia | *Plasmodium berghei* ANKA | C57BL/6 mice | Heiss *et al.,* 2018 |
| [S147]* | Artemether in nanostructured lipid carrier | Prevented recrudescence | *Plasmodium berghei* ANKA | C57BL/6 mice | Vanka *et al.,*2018 |
| [S148] | Doxycycline* | Prevented CM | *Plasmodium berghei* ANKA | C57BL/6 mice | Schmidt *et al.,* 2018 |
| [S149] | Ethanolic extract *Trichoderma stromaticum* | Improved survival prevented sequelae | *Plasmodium berghei* ANKA | C57BL/6 mice | Cariaco *et al.,* 2018 |
| [S150] | Concurrent Chikungunya infection | Improved survival Improved CM | *Plasmodium berghei* ANKA | C57BL/6 mice | Teo *et al.,*2018 |
| [S151] | Perillyl alcohol | Reduced inflammation | *Plasmodium berghei* ANKA | C57BL/6 mice | Rodriguez *et al.,* 2018 |
| [S152] | *Terminalia albida* | Reduced inflammation Reduced parasitaemia | *Plasmodium berghei* ANKA | C57BL/6 mice | Camara *et al.,*2019 |
| [S153] | Fenozyme | Increased survival Improved CM | *Plasmodium berghei* ANKA | C57BL/6 mice | Zhao *et al.,*2019 |
| [S154] | L-arginine supplementation and thromboxane synthase inhibition | Improved CM | *Plasmodium berghei* ANKA | C57BL/6 mice | Moreira *et al.,* 2019 |
| [S155] | T-cell Immunomodulatory Protein | Reduced pathology | *Plasmodium berghei* ANKA | C57BL/6 and BALB/c mice | Cui *et al.,* 2019 |
| [S156] | Coenzyme Q10 | Improved survival Improved CM Reduced inflammation | *Plasmodium berghei* ANKA | C57BL/6 | Nyariki *et al.,* 2019 |
| [S157] | Citrulline | Improved survival | *Plasmodium berghei* ANKA | C57BL/6 | Gramaglia *et al.,* 2019 |
| [S158] | PDL1 Fusion Protein | Improved survival | *Plasmodium berghei* ANKA | C57BL/6 | Wang *et al.,* 2019 |
| [S159] | Melatonin | Improved CM Reduced complications | *Plasmodium berghei* ANKA | Swiss albino mice | Ataide *et al.,* 2020 |
| [S160] | Nanotized curcumin-benzothiophene conjugate | Reduced parasitaemia Improved survival | *Plasmodium berghei* ANKA | C57BL/6 | Ghosh *et al.,* 2020 |
| [S161]* | Benzene Aminoquinolines | Reduced parasitaemia Improved survival | *Plasmodium berghei* ANKA | C57BL/6 | Srbljanović *et al.,* 2020 |
| [S162] | PD1 fusion protein | Prevented neurological sequelae | *Plasmodium berghei* ANKA | C57BL/6 | Jiang *et al.,* 2020 |
| [S163] | Mesenchymal stromal cells | Improved CM Reduced sequelae | *Plasmodium berghei* ANKA | C57BL/6 | Lima *et al.,* 2020 |
| [S164] | Tetramethylpyrazine | Improved CM | *Plasmodium berghei* ANKA | C57BL/6 | Zheng *et al.,* 2021 |
| [S165]* | Artemisone microemulsion (ME) | Prevented CM | *Plasmodium berghei* ANKA | C57BL/6 | Zech *et al.,* 2021 |
| [S166] | Whole blood transfusion | Improved survival | *Plasmodium berghei* ANKA | C57BL/6 | Gul *et al.,* 2021 |
| [S167] | Monocyte Locomotion Inhibitory Factor (MLIF) | Increased survival. Prevented neurological sequelae | *Plasmodium berghei* ANKA | C57BL/6 | Galán-Salinas *et al.,* 2021 |
| [S168]* | Artesunate-loaded nanostructured lipid carriers for intranasal administration | Reduced parasitaemia | *Plasmodium berghei* ANKA | CBA/J | Agbo *et al.,* 2021 |
| [S169] | Anti-CD47 | Reduced inflammation; Improved CM Improved survival | *Plasmodium berghei* ANKA | C57BL/6 | Torrez *et al.,* 2021 |
| [S170]* | Self-microemulsifying drug delivery system (SMEDDS) for artemisone | Improved CM | *Plasmodium berghei* ANKA | C57BL/6 | Zech *et al.,* 2021 |
| [S171] | IL-4 | Reduced parasitaemia Improved CM Increased survival | *Plasmodium berghei* ANKA | C57BL/6 | Wu *et al.,* 2021 |
| [S172] | Anti-CD146 antibody AA98 | Improved CM Increased survival | *Plasmodium berghei* ANKA | C57BL/6 | Duan *et al.,* 2021 |
| [S173] | Anti-Lt-α antibody | Increased survival Improved CM | *Plasmodium berghei* ANKA | C57BL/6 | Eeka *et al.,* 2022 |
| [S174] | α-Tocopheryloxy Acetic Acid | Improved CM | *Plasmodium berghei* ANKA | C57BL/6 | Ariefta *et al.,* 2022 |
| [S175] | Griseofulvin | Improved and prevented CM | *Plasmodium berghei* ANKA | C57BL/6 | Chandana *et al.,* 2022 |
| [S176] | Glucose-modified nanocomposite liposomes | Reduced infection rate and recurrence | *Plasmodium berghei* ANKA | C57BL/6 | Tian *et al.,* 2022 |
| [S177] | Monoterpene 1,8-cineole | Improved CM Reduced parasitaemia | *Plasmodium berghei* ANKA | C57BL/6 | Santos *et al.,* 2022 |
| [S178] | Hypothyroidism, Sirtuin 1 | Improved CM Reduced mortality | *Plasmodium berghei* ANKA | C57BL/6 | Rodriguez-Muñoz *et al.,* 2022 |
| [S179] | *Listeria monocytogenes* Inoculation | Improved CM | *Plasmodium berghei* ANKA | C57BL/6 | Yang *et al.,* 2022 |
| [S180]* | Quinoline salt derivative | Reduced parasitaemia Prevented CM | *Plasmodium falciparum* | C57BL/6 | Bezerra Bellei *et al.,* 2022 |
| [S181] | *Euterpe oleracea* fruit (Açai)-enriched diet | Improved survival Improved CM Reduced neurocognitive deficits | *Plasmodium berghei* ANKA | Swiss albino mice | Oliveira *et al.,* 2022 |
| [S182]* | Nanoparticle formulation dihydroartemisinin coated in a brain microvascular endothelial cell derived biomimetic membrane | Improved CM Improved survival | *Could not be found as publication could not be accessed | *Could not be found | Wei *et al.,* 2022 |
| [S183] | Apocynin | Improved CM Reduced long-term effects | *Plasmodium berghei* ANKA | C57BL/6 | Kumar *et al.,* 2022 |
| [S184] | 3-methyladenine bafilomycin A1 | Improved CM | *Plasmodium berghei* ANKA | C57BL/6 | Leleu *et al.,* 2022 |
| [S185] | Monoclonal antibody to pathological tau (PHF-1 mAB) | Reduced inflammation Reduced cognitive impairment | *Plasmodium berghei* ANKA | C57BL/6 | Akide Ndunge *et al.,* 2023 |
| [S186] | *Atractylodes lancea* or *Prabchompoothawe*sp remedy extracts | Combination: Improved CM Increased survival Reduced cognitive impairment | *Plasmodium berghei* ANKA | C57BL/6 mice | Plirat *et al.,* 2023 |
| [S187] | aqueous extract *Phyllanthus niruri* | Reduced parasitaemia Increased survival Reduced inflammation | *Plasmodium berghei* ANKA | C57BL/6 mice | Jeje *et al.,* 2023 |
| [S188] | Coenzyme Q10 | Reduced parasitaemia, Improved survival | *Plasmodium berghei* ANKA | C57BL6 | Nyakiri *et al.,* 2023 |
| [S189] | Whole blood transfusion | Increased survival Improved CM | *Plasmodium berghei* ANKA | C57BL6 | Gul *et al.,* 2023 |
| [S190]* | Minocycline | Reduced proinflammatory cytokines Prevented long-term cognitive decline | *Plasmodium berghei* ANKA | C57BL6 | Moreira *et al.,* 2024 |
| [S191] | Aminoguanidine | Reduced severity of CM Prevented long-term sequelae | *Plasmodium berghei* ANKA | C57BL6 | Silva *et al.,* 2024 |
| [S192] | IPI549 | Reduced inflammation Prevented CM | *Plasmodium berghei* ANKA | C57BL/6J | Jin *et al.,* 2024 |
| [S193] | Irbesartan and losartan | Improved survival Reduced inflammation | *Plasmodium berghei* ANKA | C57BL6 mice | Shaham *et al.,* 2024 |
| [S194] | Extracellular vesicle immunization | Improved survival | *Plasmodium berghei* ANKA | C57BL/6J | Lv *et al.,* 2024 |
| [S195] | Angiotensin-2 derivatives | Reduced parasitaemia Increased survival | *Plasmodium berghei* ANKA | BALB/c mice | Silva *et al.,* 2024 |
| [S196]* | Liposomal mefloquine | Improved CM | *Plasmodium berghei* ANKA | *Could not be found as publication could not be accessed | Raza *et al.,* 2024 |
| [S197] | DHA-rich fish oil | Improved CM Improved survival | *Plasmodium berghei ANKA* | C57BL/6 | Carpenter *et al.,* 2024 |

*antimalarial drugs; expected to be successful in comparison with no treatment

**Table S2**

List of clinical trials on human cerebral malaria from 1956 to 2024 – includes clinical trials on severe malaria that included patients with cerebral malaria

| Number | Intervention | Effect | Reference | Year |
| --- | --- | --- | --- | --- |
| [S198] | Urea | No benefit | Rothe 1956 | 1956 |
| [S199] | Urea | No benefit | Kingston 1971 | 1971 |
| [S200] | Dexamethasone | Harmful | Warrell *et al.,* 1982 | 1982 |
| [S201] | High dose Dexamethasone | No benefit | Hoffman *et al.,* 1988 | 1988 |
| [S202] | Low dose Phenobarbital | Fewer convulsions | White *et al.,* 1988 | 1988 |
| [S203] | Heparin or acetylsalicilic acid | No benefit | Hemmer *et al.,* 1991 | 1991 |
| [S204] | Deferoxamine | No benefit | Gordeauk *et al.,* 1992 | 1992 |
| [S205] | Immunoglobulin | Harmful | Taylor *et al.,* 1992 | 1992 |
| [S206] | Dichloroacetate | No benefit | Krishna *et al.,* 1994 | 1994 |
| [S207] | Pentoxyfylline | No benefit | Di Perri *et al.,* 1995 | 1995 |
| [S208] | Dichloroacetate | No benefit | Krishna *et al.,* 1995 | 1995 |
| [S209] | anti-TNF monoclonal antibody | No benefit | van Hensbroek *et al.,1996* | 1996 |
| [S210] | Dichloroacetate | No benefit | Krishna *et al.,* 1996 | 1996 |
| [S211] | Pentoxyfylline | No benefit | Hemmer *et al.,* 1997 | 1997 |
| [S212] | Pentoxyfylline | No benefit | Looareesuwan *et al.,* 1998 | 1998 |
| [S213] | Deferoxamine | No benefit | Thuma *et al.,* 1998 | 1998 |
| [S214] | High dose phenobarbital | Harmful | Crawley *et al.,* 2000 | 2000 |
| [S215] | Deferiprone | No benefit | Mohanty *et al.*, 2002 | 2002 |
| [S216] | N-Acetylcysteine | beneficial | Watt *et al.,* 2002 | 2002 |
| [S217] | Pentoxyfylline | No benefit | Das *et al.,* 2003 | 2003 |
| [S218] | N-Acetylcysteine | No benefit | Treeprasertuk *et al.,* 2003 | 2003 |
| [S219] | Dichloroacetate | Reduced blood lactate | Agbenyega *et al.,* 2003 | 2003 |
| [S220] | Curdlan sulphate | No benefit | Havlik *et al,* 2005 | 2005 |
| [S221] | Albumin | No significant benefit | Maitland et al.,2005 | 2005 |
| [S222] | Gelofusine | No significant benefit | Akech *et al.,* 2006 | 2006 |
| [S223] | Mannitol | No benefit | Namutangula *et al.,* 2007 | 2007 |
| [S224] | N-Acetylcysteine | No benefit | Charunwatthana *et al.,* 2009 | 2009 |
| [S225] | Erythropoetin | No benefit | Picot et al., 2009 | 2009 |
| [S226] | Pentoxyfylline | Harmful | Lell *et al.,* 2005 | 2010 |
| [S227] | Activated charcoal | No benefit | De Souza et al., 2010 | 2010 |
| [S228] | Mannitol | Harmful | Mohanty *et al.,* 2011 | 2011 |
| [S229] | Bolus high volume albumin/saline | Harmful | Maitland *et al.,* 2011 | 2011 |
| [S230] | Early enteral feeding | Harmful | Maude et al., 2011 | 2011 |
| [S231] | Vitamin A | No benefit | Mwanga-Amumpaire *et al*.,2012 | 2012 |
| [S232] | Levamisole | No benefit | Maude *et al.,* 2014 | 2014 |
| [S233] | Nitric Oxide | No benefit | Hawkes *et al.,* 2015 | 2015 |
| [S234] | Nitric Oxide | No benefit | Mwanga-Amumpaire *et al., 2015* | 2015 |
| [S235] | Delayed iron therapy | No benefit | Cusick *et al.,* 2016 | 2016 |
| [S236] | Delayed iron therapy | No benefit | Cusick *et al.,* 2020 | 2020 |
| [S237] | Delayed iron therapy | No benefit | Ssemata *et al.,* 2020 | 2020 |
| [S238] | Aggressive antipyretic therapy | Concerns over harm | Birbeck et al., 2024 | 2024 |

**Supplementary References by year and first author for the laboratory trials in the murine model of cerebral malaria**

1. Grau, G.E. *et al*. (1987) Tumor necrosis factor (cachectin) as an essential mediator in murine cerebral malaria. *Science.* 237, 1210-1212
2. Grau, G.E. *et al*. (1987) Prevention of murine cerebral malaria by low-dose cyclosporin A. *Immunology*  61 , 521-525
3. Grau, G.E. *et al*. (1988) Prevention of experimental cerebral malaria by anticytokine antibodies. Interleukin 3 and granulocyte macrophage colony-stimulating factor are intermediates in increased tumor necrosis factor production and macrophage accumulation. *J. Exp. Med*. 168, 1499-1504
4. Thumwood, C.M. *et al*. (1989) Antioxidants can prevent cerebral malaria in *Plasmodium berghei*-infected mice. *Br. J. Exp. Pathol.* 70, 293-303
5. Grau G.E. *et al*. (1989) Monoclonal antibody against interferon gamma can prevent experimental cerebral malaria and its associated overproduction of tumor necrosis factor. *Proc. Natl. Acad. Sci. U. S. A.*  86, 5572-5574
6. Curfs, J.H. *et al*. (1990) Low dosages of interleukin 1 protect mice against lethal cerebral malaria. *J. Exp. Med.* 172, 1287-1291
7. Grau, G.E. *et al*. (1987) Late administration of monoclonal antibody to leukocyte function-antigen 1 abrogates incipient murine cerebral malaria. *Eur. J. Immunol.* 21, 2265-2267
8. Kremsner, P.G. *et al*. (1991) Pentoxifylline prevents murine cerebral malaria. *J. Infect. Dis*. 164, 605-608
9. Sliwa, K. *et al*. (1991) Prevention of murine cerebral malaria by a stable prostacyclin analog. *Infect. Immun.* 59, 3846-3848
10. Blok, W.L. *et al*. (1992) Dietary fish-oil supplementation in experimental gram-negative infection and in cerebral malaria in mice. *J. Infect. Dis*. 165, 898-903
11. Blok, W.L. et al. (1992) Dietary fish-oil supplementation in experimental gram-negative infection and in cerebral malaria in mice. *J. Infect. Dis*. 165, 898-903
12. Hunt, N.H. *et al*. (1993) Amelioration of murine cerebral malaria by dietary restriction. *Parasitology* 107, 471-476
13. Eckwalanga, M. *et al*. (1994) Murine AIDS protects mice against experimental cerebral malaria: down-regulation by interleukin 10 of a T-helper type 1 CD4+ cell-mediated pathology. *Proc. Natl. Acad. Sci. U. S. A.* 91, 8097-8101
14. Levander, O.A. *et al*. (1995) Protection against murine cerebral malaria by dietary-induced oxidative stress. *J. Parasitol.* 81, 99-103
15. Neill, A.L. and Hunt, N.H. (1995) Effects of endotoxin and dexamethasone on cerebral malaria in mice. *Parasitology* 111, 443-454
16. Moumaris, M. *et al*. (1995) Effect of fatty acid treatment in cerebral malaria-susceptible and non-susceptible strains of mice. *J. Parasitol*. 81, 997-999
17. Yan, Y. *et al*. (1997) Down-regulation of murine susceptibility to cerebral malaria by inoculation with third-stage larvae of the filarial nematode *Brugia pahangi*. *Parasitology* 114 , 333-338
18. Hermsen, C. *et al*. (1997) Depletion of CD4+ or CD8+ T-cells prevents *Plasmodium berghei* induced cerebral malaria in end-stage disease. *Parasitology* 114 , 7-12
19. Kossodo, S. *et al*. (1997) Interleukin-10 modulates susceptibility in experimental cerebral malaria. *Immunology*. 91, 536-544
20. Hermsen, C.C. *et al*. (1998) Convulsions due to increased permeability of the blood-brain barrier in experimental cerebral malaria can be prevented by splenectomy or anti-T cell treatment. *J. Infect. Dis.* 178, 1225-1227
21. Postma, N.S. *et al*. (1999) Thiolated recombinant human tumor necrosis factor-alpha protects against *Plasmodium berghei* K173-induced experimental cerebral malaria in mice. *Antimicrob. Agents. Chemother.* 43, 1027-1033
22. Postma, N.S. *et al*. (1999) Treatment with recombinant human tumor necrosis factor-alpha reduces parasitemia and prevents *Plasmodium berghei* K 173 - Induced experimental cerebral malaria in mice. *Parasitology* 118, 7-15
23. Rae, C. *et al*. (2000) Dichloroacetate (DCA) reduces brain lactate but increases brain glutamine in experimental cerebral malaria: a 1H-NMR study. *Redox Rep.* 5, 141-143
24. Schofield, L. (2002) Synthetic GPI as a candidate anti-toxic vaccine in a model of malaria. *Nature* 418, 785-789
25. Sun, G. *et al*. (2003) Inhibition of platelet adherence to brain microvasculature protects against severe *Plasmodium berghei* malaria. *Infect. Immun* 71, 6553-61.
26. Sun, G. et al. (2003) Inhibition of platelet adherence to brain microvasculature protects against severe *Plasmodium berghei* malaria. Infect. Immun 71, 6553-61.
27. Ball, H.J. *et al*. (2004) Cyclooxygenase-2 in the pathogenesis of murine cerebral malaria. *J. Infect. Dis.* 189, 751-758
28. Clark, C.J. *et al*. (2005) Prolonged survival of a murine model of cerebral malaria by kynurenine pathway inhibition. *Infect. Immun.* 73, 5249-5251
29. Muniz-Junqueira, M.I. *et al*. (2005) Thalidomide influences the function of macrophages and increases the survival of *Plasmodium berghei*-infected CBA mice. *Acta .Trop.* 94, 128-138
30. Kaiser, K. *et al*. (2006) Recombinant human erythropoietin prevents the death of mice during cerebral malaria. *J. Infect. Dis*. 193, 987-995
31. Gramaglia, I. *et al*. (2006) Low nitric oxide bioavailability contributes to the genesis of experimental cerebral malaria. *Nat. Med.* 12, 1417-1422
32. Lepenies, B. *et al*. (2007) Ligation of B and T lymphocyte attenuator prevents the genesis of experimental cerebral malaria. *J. Immunol.* 179, 4093-4100
33. Pamplona, A. *et al*. (2007) Heme oxygenase-1 and carbon monoxide suppress the pathogenesis of experimental cerebral malaria. *Nat. Med.* 13, 703-710
34. Vigário, A.M. *et al*. (2007) Recombinant human IFN-alpha inhibits cerebral malaria and reduces parasite burden in mice. *J. Immunol.* 178, 6416-6425
35. Amante, F.H. *et al*. (2007) A role for natural regulatory T cells in the pathogenesis of experimental cerebral malaria. *Am. J. Pathol.* 171, 548-559
36. Randall, L.M. *et al*. (2008) Common strategies to prevent and modulate experimental cerebral malaria in mouse strains with different susceptibilities. *Infect. Immun.* 76, 3312-3320
37. Specht, S. *et al*. (2008) The guanylhydrazone CNI-1493: an inhibitor with dual activity against malaria-inhibition of host cell pro-inflammatory cytokine release and parasitic deoxyhypusine synthase. *Parasitol. Res.* 102, 1177-1184
38. Patel, S.N. *et al*. (2008) C5 deficiency and C5a or C5aR blockade protects against cerebral malaria. *J. Exp. Med.* 205, 1133-1143
39. Penet, M.F. *et al*. (2008) Protection against cerebral malaria by the low-molecular-weight thiol pantethine. *Proc. Natl. Acad. Sci. U. S. A.* 105, 1321-1326
40. Kobbe, R. *et al*. (2008) Simvastatin treatment shows no effect on the incidence of cerebral malaria or parasitemia during experimental malaria. *Antimicrob. Agents. Chemother.* 52, 1583-1584
41. Srivastava, K. *et al*. (2008) Platelet factor 4 mediates inflammation in experimental cerebral malaria. *Cell. Host Microbe.* 4, 179-187
42. Srivastava, K. et al. (2008) Platelet factor 4 mediates inflammation in experimental cerebral malaria. *Cell. Host Microbe*. 4, 179-187
43. Helmers, A.J. *et al*. (2008) Failure of two distinct anti-apoptotic approaches to reduce mortality in experimental cerebral malaria. *Am. J. Trop. Med. Hyg.* 79, 823-825
44. Robert V. *et al*. (2008) Malaria and obesity: obese mice are resistant to cerebral malaria. *Malar. J.* 7, 81
45. Blanco, Y.C. *et al*. (2008) Hyperbaric oxygen prevents early death caused by experimental cerebral malaria. *PLoS One.* 3, e3126
46. Lackner, P. *et al*. (2009) Glatiramer acetate reduces the risk for experimental cerebral malaria: a pilot study, Part A. *Malar. J.* 2009*;* 8, 36
47. Nie, C.Q. *et al*. (2009) IP-10-mediated T cell homing promotes cerebral inflammation over splenic immunity to malaria infection. *PLoS Pathog.* 2009*;* 5, e1000369
48. Serghides, L. *et al*. (2009) Rosiglitazone modulates the innate immune response to *Plasmodium falciparum* infection and improves outcome in experimental cerebral malaria. *J. Infect. Dis.* 2009; 199, 1536-154
49. Waknine-Grinberg JH,et al. (2010) Artemisone effective against murine cerebral malaria. *Malar J*. 2010; 9:227.
50. Specht S, et al. (2010) Filaria-induced IL-10 suppresses murine cerebral malaria. *Microbes Infect*. 2010;12(8-9):635-642.
51. Waknine-Grinberg JH, et al. (2010) Modulation of cerebral malaria by fasudil and other immune-modifying compounds. *Exp Parasitol*. 2010;125(2):141-146.
52. Waknine-Grinberg JH, et al. (2010) *Schistosoma mansoni* infection reduces the incidence of murine cerebral malaria. *Malar J*. 2010;9:5.
53. Herbas MS, et al. (2010) alpha-Tocopherol transfer protein inhibition is effective in the prevention of cerebral malaria in mice. *Am J Clin Nutr*. 2010;91(1):200-207.
54. Cabrales P, et al. (2010) Murine cerebral malaria is associated with a vasospasm-like microcirculatory dysfunction, and survival upon rescue treatment is markedly increased by nimodipine. *Am J Pathol*. 2010;176(3):1306-1315.
55. Hein-Kristensen L, et al. (2010) Simultaneous administration of vitamin A and DTP vaccine modulates the immune response in a murine cerebral malaria model. *Scand J Immunol*. 2010;72(4):302-308.
56. de Souza JB, et al. (2010) Oral activated charcoal prevents experimental cerebral malaria in mice and in a randomized controlled clinical trial in man did not interfere with the pharmacokinetics of parenteral artesunate. *PLoS One*. 2010;5(4):e9867.
57. Haque A, et al. (2010) CD4+ natural regulatory T cells prevent experimental cerebral malaria via CTLA-4 when expanded in vivo. *PLoS Pathog*. 2010;6(12):e1001221.
58. Serghides L, et al. (2011) Inhaled nitric oxide reduces endothelial activation and parasite accumulation in the brain, and enhances survival in experimental cerebral malaria. *PLoS One*. 2011;6(11):e27714.
59. Wang HZ, et al . (2011) Hepcidin is regulated during blood-stage malaria and plays a protective role in malaria infection. *J Immunol*. 2011;187(12):6410-6416.
60. Finney CA, et al. (2011) S1P is associated with protection in human and experimental cerebral malaria. *Mol Med*. 2011;17(7-8):717-725.
61. Cabrales P, et al. (2011) Nitric oxide protection against murine cerebral malaria is associated with improved cerebral microcirculatory physiology. *J Infect Dis*. 2011;203(10):1454-1463.
62. Bertinaria M, et al. (2011) Amodiaquine analogues containing NO-donor substructures: synthesis and their preliminary evaluation as potential tools in the treatment of cerebral malaria. *Eur J Med Chem*. 2011;46(5):1757-1767.
63. Franklin BS, et al. (2011) Therapeutical targeting of nucleic acid-sensing Toll-like receptors prevents experimental cerebral malaria. *Proc Natl Acad Sci U S A*. 2011;108(9):3689-3694.
64. Morrell CN, et al. (2011) Beta interferon suppresses the development of experimental cerebral malaria. *Infect Immun*. 2011;79(4):1750-1758.
65. Clemmer L, et al. (2011) Artemether and artesunate show the highest efficacies in rescuing mice with late-stage cerebral malaria and rapidly decrease leukocyte accumulation in the brain. *Antimicrob Agents Chemother*. 2011;55(4):1383-1390.
66. Gerald NJ, et al. (2011) Protection from experimental cerebral malaria with a single dose of radiation-attenuated, blood-stage *Plasmodium berghei* parasites. *PLoS One*. 2011;6(9):e24398.
67. Tamura T, et al. (2011) Prevention of experimental cerebral malaria by Flt3 ligand during infection with *Plasmodium berghei* ANKA. *Infect Immun*. 2011;79(10):3947-3956.
68. Zanini GM, et al. (2011) Exogenous nitric oxide decreases brain vascular inflammation, leakage and venular resistance during *Plasmodium berghei* ANKA infection in mice. *J Neuroinflammation*. 2011;8:66.
69. Reis PA, et al. (2012) Statins decrease neuroinflammation and prevent cognitive impairment after cerebral malaria. *PLoS Pathog*. 2012;8(12): e1003099.
70. Dai M, et al. (2012) Altered regulation of Akt signaling with murine cerebral malaria, effects on long-term neuro-cognitive function, restoration with lithium treatment. *PLoS One*. 2012;7(10): e44117.
71. Dai M, et al. (2012) The novel ETA receptor antagonist HJP-272 prevents cerebral microvascular hemorrhage in cerebral malaria and synergistically improves survival in combination with an artemisinin derivative. *Life Sci*. 2012; 91(13-14):687-692.
72. Hempel C, et al . (2012) Erythropoietin treatment alleviates ultrastructural myelin changes induced by murine cerebral malaria. *Malar J*. 2012;11:216.
73. Zhu X, et al. (2012) Targeting Toll-like receptors by chloroquine protects mice from experimental cerebral malaria. *Int Immunopharmacol*. 2012;13(4):392-397.
74. Zanini GM, et al. (2012) S-nitrosoglutathione prevents experimental cerebral malaria. *J Neuroimmune Pharmacol*. 2012;7(2):477-487.
75. Martins YC, et al. (2012) Efficacy of different nitric oxide-based strategies in preventing experimental cerebral malaria by *Plasmodium berghei* ANKA. *PLoS One*. 2012;7(2): e32048.
76. Souraud JB, et al. (2012) Atorvastatin treatment is effective when used in combination with mefloquine in an experimental cerebral malaria murine model. *Malar J*. 2012; 11:13..
77. Pena AC, et al. (2012) A novel carbon monoxide-releasing molecule fully protects mice from severe malaria. *Antimicrob Agents Chemother*. 2012;56(3):1281-1290.
78. Guo J, et al. (2012) Synthesis of artemiside and its effects in combination with conventional drugs against severe murine malaria. *Antimicrob Agents Chemother*. 2012;56(1):163-173.
79. Miranda AS, et al. (2013) Further evidence for an anti-inflammatory role of artesunate in experimental cerebral malaria. *Malar J*. 2013;12:388.
80. Waknine-Grinberg JH, et al. (2013) Glucocorticosteroids in nano-sterically stabilized liposomes are efficacious for elimination of the acute symptoms of experimental cerebral malaria. *PLoS One*. 2013; 8(8):e72722.
81. Dormoi J, et al. (2013) Improvement of the efficacy of dihydroartemisinin with atorvastatin in an experimental cerebral malaria murine model. *Malar J*. 2013;12:302.
82. Bedri S, et al. (2013) Azadirachta indica ethanolic extract protects neurons from apoptosis and mitigates brain swelling in experimental cerebral malaria. *Malar J*. 2013; 12:298.
83. Orjuela-Sánchez P, et al. (2013) Transdermal glyceryl trinitrate as an effective adjunctive treatment with artemether for late-stage experimental cerebral malaria. *Antimicrob Agents Chemother*. 2013; 57(11):5462-5471.
84. Wilson NO, et al. (2013) Pharmacologic inhibition of CXCL10 in combination with anti-malarial therapy eliminates mortality associated with murine model of cerebral malaria. *PLoS One*. 2013; 8(4):e60898.
85. Martins YC, et al. (2013) Slow and continuous delivery of a low dose of nimodipine improves survival and electrocardiogram parameters in rescue therapy of mice with experimental cerebral malaria. *Malar J*. 2013; 12:138.
86. Dormoi J, et al. (2013) Efficacy of proveblue (methylene blue) in an experimental cerebral malaria murine model. *Antimicrob Agents Chemother*. 2013;57(7):3412-3414.
87. Dormoi J, et al. (2013) Impact of methylene blue and atorvastatin combination therapy on the apparition of cerebral malaria in a murine model. *Malar J*. 2013; 12:127.
88. DellaValle B, et al. (2013) Investigation of hydrogen sulfide gas as a treatment against *P. falciparum*, murine cerebral malaria, and the importance of thiolation state in the development of cerebral malaria. *PLoS One*. 2013; 8(3):e59271.
89. Anand SS, et al. (2013) The specific, reversible JNK inhibitor SP600125 improves survivability and attenuates neuronal cell death in experimental cerebral malaria (ECM). *Parasitol Res*. 2013; 112(5):1959-1966.
90. Karlsson M, et al. (2013) Brain mitochondrial function in a murine model of cerebral malaria and the therapeutic effects of rhEPO. *Int J Biochem Cell Biol*. 2013;45(1):151-155.
91. Wei X, et al. (2014) Erythropoietin protects against murine cerebral malaria through actions on host cellular immunity. *Infect Immun.* 2014;82(1):165-173.
92. Shryock N, et al. Lipoxin A₄ and 15-epi-lipoxin A₄ protect against experimental cerebral malaria by inhibiting IL-12/IFN-γ in the brain. *PLoS One*. 2013; 8(4):e61882.
93. Marijon A, et al. (2014) Efficacy of intranasal administration of artesunate in experimental cerebral malaria. *Malar J*. 2014; 13:501.
94. Recuenco FC, Takano R, Chiba S, et al. Lambda-carrageenan treatment exacerbates the severity of cerebral malaria caused by Plasmodium berghei ANKA in BALB/c mice. *Malar J*. 2014;13:487.
95. Guo J, et al. (2014) Reduction of experimental cerebral malaria and its related proinflammatory responses by the novel liposome-based β-methasone nanodrug. *Biomed Res Int*. 2014;2014:292471.
96. He X, et al. (2014) Vitamin D inhibits the occurrence of experimental cerebral malaria in mice by suppressing the host inflammatory response. *J Immunol*. 2014;193(3):1314-1323.
97. Guiguemde WA, et al. (2014) Treatment of Murine Cerebral Malaria by Artemisone in Combination with Conventional Antimalarial Drugs: Antiplasmodial Effects and Immune Responses. *Antimicrob Agents Chemother* 2014; 58: 4745-54.
98. Serghides L, et al. (2014) PPARγ agonists improve survival and neurocognitive outcomes in experimental cerebral malaria and induce neuroprotective pathways in human malaria. *PLoS Pathog*. 2014;10(3): e1003980.
99. Francischetti IM, et al. (2014) Tempol, an intracellular antioxidant, inhibits tissue factor expression, attenuates dendritic cell function, and is partially protective in a murine model of cerebral malaria. *PLoS One*. 2014;9(2):e87140
100. Solomon W, et al. (2014) Neuregulin-1 attenuates mortality associated with experimental cerebral malaria. *J Neuroinflammation* **11**, 9.
101. El-Assaad F, et al. Potential efficacy of citicoline as adjunct therapy in treatment of cerebral malaria. *Antimicrob Agents Chemother*. 2014;58(1):602-605.
102. Gordon EB, et al. (2015) Targeting glutamine metabolism rescues mice from late-stage cerebral malaria. *Proc Natl Acad Sci U S A*. 2015;112(42):13075-13080.
103. Canavese M, Crisanti A. Vascular endothelial growth factor (VEGF) and lovastatin suppress the inflammatory response to *Plasmodium berghei* infection and protect against experimental cerebral malaria. *Pathog Glob Health*. 2015;109(6):266-274.
104. Canavese M, et al. (2015) VEGF and LPS synergistically silence inflammatory response to *Plasmodium berghei* infection and protect against cerebral malaria. *Pathog Glob Health*. 2015;109(6):255-265.
105. Gallego-Delgado J , et al. (2015) Angiotensin II Moderately Decreases Plasmodium Infection and Experimental Cerebral Malaria in Mice. *PLoS One*. 2015;10(9):e0138191. Published 2015 Sep 16.
106. Bertinaria M, et al. (2015) NO-Donor Dihydroartemisinin Derivatives as Multitarget Agents for the Treatment of Cerebral Malaria. *J Med Chem*. 2015;58(19):7895-7899.
107. Howland SW, et al. (2015) Investigating proteasome inhibitors as potential adjunct therapies for experimental cerebral malaria. *Parasite Immunol*. 2015;37(11):599-604.
108. Leitner DF, et al. (2015) The HFE genotype and a formulated diet controlling for iron status attenuate experimental cerebral malaria in mice. *Int J Parasitol*. 2015;45(12):797-808.
109. Dende C, et al. (2015) Simultaneously targeting inflammatory response and parasite sequestration in brain to treat Experimental Cerebral Malaria. *Sci Rep*. 2015;5:12671.
110. Gordon EB, et al. Inhibiting the Mammalian target of rapamycin blocks the development of experimental cerebral malaria. *mBio*. 2015;6(3): e00725.
111. Yalcin O, et al. (2015) From METS to malaria: RRx-001, a multi-faceted anticancer agent with activity in cerebral malaria. *Malar J*. 2015; 14:218.
112. Souza MC, et al. (2015) Mesenchymal stromal cell therapy attenuated lung and kidney injury but not brain damage in experimental cerebral malaria. *Stem Cell Res Ther*. 2015;6(1):102.
113. Xu H, et al. (2015) L-arginine exacerbates experimental cerebral malaria by enhancing pro-inflammatory responses. *Tohoku J Exp Med*. 2015;236(1):21-31.
114. van den Ham KM, et al. (2015) Iron prevents the development of experimental cerebral malaria by attenuating CXCR3-mediated T cell chemotaxis. *PLoS One*. 2015;10(3):e0118451.
115. Mejia P, et al. (2015) Dietary restriction protects against experimental cerebral malaria via leptin modulation and T-cell mTORC1 suppression. *Nat Commun*. 2015; 6: 6050.
116. Campos AC, et al. (2015) Cannabidiol increases survival and promotes rescue of cognitive function in a murine model of cerebral malaria. *Neuroscience*. 2015;289:166-180.
117. Val CH, et al. (2015) Effect of mushroom *Agaricus blazei* on immune response and development of experimental cerebral malaria. *Malar J*. 2015; 14:311.
118. Meadows DN, et al. (2015) High Dietary Folate in Mice Alters Immune Response and Reduces Survival after Malarial Infection. *PLoS One*. 2015;10(11):e0143738.
119. Prabhu P, et al. (2016) Nanostructured lipid carriers of artemether-lumefantrine combination for intravenous therapy of cerebral malaria. *Int J Pharm*. 2016;513(1-2):504-517.
120. Dwivedi H, et al. (2016) Potential cerebral malaria therapy: intramuscular arteether and vitamin D co-administration. *Parasitology*. 2016;143(12):1557-1568.
121. Moradin N, et al. (2016) Cysteamine broadly improves the anti-plasmodial activity of artemisinins against murine blood stage and cerebral malaria. *Malar J*. 2016;15(1):260.
122. Martins YC, et al. (2016) Endothelin-1 Treatment Induces an Experimental Cerebral Malaria-Like Syndrome in C57BL/6 Mice Infected with *Plasmodium berghei* NK65. *Am J Pathol*. 2016;186(11):2957-2969.
123. Crowley VM, et al. (2017) Synthetic oleanane triterpenoids enhance blood brain barrier integrity and improve survival in experimental cerebral malaria. *Malar J*. 2017;16(1):463.
124. Mejia P, et al. (2017) A single rapamycin dose protects against late-stage experimental cerebral malaria via modulation of host immunity, endothelial activation and parasite sequestration. *Malar J*. 2017; 16(1):455.
125. Dende C, et al. (2017) Nanocurcumin is superior to native curcumin in preventing degenerative changes in Experimental Cerebral Malaria. *Sci Rep*. 2017;7(1):10062.
126. van den Ham KM, et al. (2017) Protein Tyrosine Phosphatase Inhibition Prevents Experimental Cerebral Malaria by Precluding CXCR3 Expression on T Cells. *Sci Rep*. 2017;7(1):5478.
127. Wu X, et al. (2017) Small molecule-based inhibition of MEK1/2 proteins dampens inflammatory responses to malaria, reduces parasite load, and mitigates pathogenic outcomes. *J Biol Chem*. 2017;292(33):13615-13634.
128. Odhiambo OC, et al. (2017) Efficacy and safety evaluation of a novel trioxaquine in the management of cerebral malaria in a mouse model. *Malar J*. 2017;16(1):268.
129. Jiang P, et al. (2017) Hydrogen sulfide protects against the development of experimental cerebral malaria in a C57BL/6 mouse model. *Mol Med Rep*. 2017;16(2):2045-2050.
130. Du Y, et al. (2017) Artesunate and erythropoietin synergistically improve the outcome of experimental cerebral malaria. *Int Immunopharmacol*. 2017;48:219-230.
131. Mubaraki MA, et al. (2017) Oxidative stress and genes regulation of cerebral malaria upon Zizyphus spina-christi treatment in a murine model. *Microb Pathog*. 2017;107:69-74.
132. Golenser J, et al. (2017) Controlled release of artemisone for the treatment of experimental cerebral malaria. *Parasit Vectors*. 2017;10(1):117.
133. Roussilhon C, et al*.* (2017) The antimicrobial molecule trappin-2/elafin has anti-parasitic properties and is protective *in vivo* in a murine model of cerebral malaria. *Sci Rep* 2017; 7: 42243 .
134. Apoorv TS, Babu PP. (2017) Minocycline prevents cerebral malaria, confers neuroprotection and increases survivability of mice during *Plasmodium berghei* ANKA infection. *Cytokine*. 2017; 90:113-123.
135. Bao LQ, et al. (2017) Tacrolimus prevents murine cerebral malaria. *Immunology*. 2017;150(2):155-161.
136. de Miranda AS, et al. (2017) A Neuroprotective Effect of the Glutamate Receptor Antagonist MK801 on Long-Term Cognitive and Behavioral Outcomes Secondary to Experimental Cerebral Malaria. *Mol Neurobiol*. 2017;54(9):7063-7082.
137. Riggle BA, et al. (2018) MRI demonstrates glutamine antagonist-mediated reversal of cerebral malaria pathology in mice. *Proc Natl Acad Sci U S A*. 2018;115(51):E12024-E12033.
138. Ong PK, et al. (2018) Reversal of cerebrovascular constriction in experimental cerebral malaria by L-arginine. *Sci Rep*. 2018;8(1):15957. Published 2018 Oct 29.
139. Wu B, et al. (2018) Oral administration of vitamin D and importance in prevention of cerebral malaria. *Int Immunopharmacol*. 2018; 64: 356-363.
140. Jiang XH, et al. (2018)  *Zhongguo Zhong Yao Za Zhi*. 2018; 43(15): 3051-3057.
141. Kume A, et al. (2018) α-Tocopheryl succinate-suppressed development of cerebral malaria in mice. *Parasitol Res*. 2018;117(10):3177-3182.
142. Strangward P, et al. (2018) Targeting the IL33-NLRP3 axis improves therapy for experimental cerebral malaria. *Proc Natl Acad Sci U S A*. 2018;115(28):7404-7409.
143. Liu M, et al. (2018) Neuregulin-1 attenuates experimental cerebral malaria (ECM) pathogenesis by regulating ErbB4/AKT/STAT3 signaling. *J Neuroinflammation*. 2018;15(1):104.
144. Burrack KS, et al. (2018) Interleukin-15 Complex Treatment Protects Mice from Cerebral Malaria by Inducing Interleukin-10-Producing Natural Killer Cells. *Immunity*. 2018;48(4):760-772.e4.
145. Langlais D, et al. (2018) Rocaglates as dual-targeting agents for experimental cerebral malaria. *Proc Natl Acad Sci U S A*. 2018;115(10): E2366-E2375.
146. Heiss K, et al. (2018) Protection from experimental cerebral malaria with a single intravenous or subcutaneous whole-parasite immunization. *Sci Rep*. 2018;8(1):3085.
147. Vanka R, et al. (2018) Ameliorating the in vivo antimalarial efficacy of artemether using nanostructured lipid carriers. *J Microencapsul*. 2018;35(2):121-136.
148. Schmidt KE, et al. (2018) Doxycycline inhibits experimental cerebral malaria by reducing inflammatory immune reactions and tissue-degrading mediators. *PLoS One*. 2018;13(2): e0192717.
149. Cariaco Y, et al. (2018) Ethanolic extract of the fungus *Trichoderma stromaticum* decreases inflammation and ameliorates experimental cerebral malaria in C57BL/6 mice. *Sci Rep* **8**, 1547 (2018).
150. Teo TH, et al. (2018) Co-infection with Chikungunya virus alters trafficking of pathogenic CD8^+^ T cells into the brain and prevents *Plasmodium*-induced neuropathology. *EMBO Mol Med*. 2018;10(1):121-138.
151. Rodriguez AAM, et al. (2018) Perillyl alcohol exhibits in vitro inhibitory activity against *Plasmodium falciparum* and protects against experimental cerebral malaria. *Int J Antimicrob Agents*. 2018;51(3):370-377.
152. Camara A, et al. (2019) *Terminalia albida* treatment improves survival in experimental cerebral malaria through reactive oxygen species scavenging and anti-inflammatory properties. *Malar J*. 2019;18(1):431.
153. Zhao S, et al. (2019) Fenozyme Protects the Integrity of the Blood-Brain Barrier against Experimental Cerebral Malaria. *Nano Lett*. 2019;19(12):8887-8895.
154. Moreira AS, et al. (2019) L-arginine supplementation and thromboxane synthase inhibition increases cerebral blood flow in experimental cerebral malaria. *Sci Rep*. 2019;9(1):13621.
155. Cui A, et al. (2019) Characterization of *Plasmodium berghei* Homologues of T-cell Immunomodulatory Protein as a New Potential Candidate for Protecting against Experimental Cerebral Malaria. *Korean J Parasitol*. 2019;57(2):101-115.
156. Nyariki JN, et al. (2019) Oral administration of Coenzyme Q_10_ protects mice against oxidative stress and neuro-inflammation during experimental cerebral malaria. *Parasitol Int*. 2019;71:106-120.
157. Gramaglia I, et al. (2019) Citrulline protects mice from experimental cerebral malaria by ameliorating hypoargininemia, urea cycle changes and vascular leak. *PLoS One*. 2019;14(3):e0213428.
158. Wang J, et al. (2019) PDL1 Fusion Protein Protects Against Experimental Cerebral Malaria via Repressing Over-Reactive CD8^+^ T Cell Responses. *Front Immunol*. 2019;9:3157.
159. Ataide BJA, et al. (2020) Melatonin Prevents Brain Damage and Neurocognitive Impairment Induced by *Plasmodium Berghei* ANKA Infection in Murine Model of Cerebral Malaria. *Front Cell Infect Microbiol*. 2020;10:541624.
160. Ghosh A, Banerjee T. (2020) Nanotized curcumin-benzothiophene conjugate: A potential combination for treatment of cerebral malaria. *IUBMB Life*. 2020;72(12):2637-2650.
161. Srbljanović J, et al. (2020) Aminoquinolines afford resistance to cerebral malaria in susceptible mice. *J Glob Antimicrob Resist*. 2020;23:20-25.
162. Jiang X, et al. (2020) Synergistic Effect of Combined Artesunate and Tetramethylpyrazine in Experimental Cerebral Malaria. *ACS Infect Dis*. 2020;6(9):2400-2409.
163. Lima MN, et al. (2020) Mesenchymal stromal cells protect against vascular damage and depression-like behavior in mice surviving cerebral malaria. *Stem Cell Res Ther*. 2020; 11(1):367.
164. Zheng Z, et al. (2020) Artesunate and Tetramethylpyrazine Exert Effects on Experimental Cerebral Malaria in a Mechanism of Protein *S*-Nitrosylation. *ACS Infect Dis*. 2021;7(10):2836-2849.
165. Zech J, et al. (2021) Transdermal delivery of artemisinins for treatment of pre-clinical cerebral malaria. *Int J Parasitol Drugs Drug Resist*. 2021;16:148-154.
166. Gul S, et al. (2021) Whole blood transfusion improves vascular integrity and increases survival in artemether-treated experimental cerebral malaria. *Sci Rep*. 2021;11(1):12077.
167. Galán-Salinas A, et al. (2021) Monocyte Locomotion Inhibitory Factor confers neuroprotection and prevents the development of murine cerebral malaria. *Int Immunopharmacol*. 2021;97:107674.
168. Agbo CP, et al. (2021) Intranasal artesunate-loaded nanostructured lipid carriers: A convenient alternative to parenteral formulations for the treatment of severe and cerebral malaria. *J Control Release*. 2021;334:224-236.
169. Torrez Dulgeroff LB, et al. (2021) CD47 blockade reduces the pathologic features of experimental cerebral malaria and promotes survival of hosts with *Plasmodium* infection. *Proc Natl Acad Sci U S A*. 2021;118(11):e1907653118.
170. Zech J, et al. (2021) Efficient Treatment of Experimental Cerebral Malaria by an Artemisone-SMEDDS System: Impact of Application Route and Dosing Frequency. *Antimicrob Agents Chemother*. 2021;65(4):e02106-20.
171. Wu X, et al. (2021) IL-4 Treatment Mitigates Experimental Cerebral Malaria by Reducing Parasitemia, Dampening Inflammation, and Lessening the Cytotoxicity of T Cells. *J Immunol*. 2021;206(1):118-131.
172. Duan H, et al. (2021) Targeting the CD146/Galectin-9 axis protects the integrity of the blood-brain barrier in experimental cerebral malaria. *Cell Mol Immunol*. 2021;18(10):2443-2454.
173. Eeka P, Phanithi PB. (2022) Lymphotoxin-α Orchestrate Hypoxia and Immune factors to Induce Experimental Cerebral Malaria: Inhibition Mitigates Pathogenesis, Neurodegeneration, and Increase Survival. *J Mol Neurosci*. 2022;72(12):2425-2439.
174. Ariefta NR, et al. (2022) Effect of α-Tocopheryloxy Acetic Acid on the Infection of Mice with *Plasmodium berghei* ANKA In Vivo and Humans with P. falciparum In Vitro. *Acta Parasitol*. 2022;67(4):1514-1520.
175. Chandana M, et al. (2022) Malaria parasite heme biosynthesis promotes and griseofulvin protects against cerebral malaria in mice. *Nat Commun*. 2022;13(1):4028.
176. Tian Y, et al. (2022) Establishment and evaluation of glucose-modified nanocomposite liposomes for the treatment of cerebral malaria. *J Nanobiotechnology*. 2022;20(1):318.
177. Santos ECD, et al. (2022) The monoterpene 1,8-cineole prevents cerebral edema in a murine model of severe malaria. *PLoS One*. 2022;17(5):e0268347.
178. Rodriguez-Muñoz D, et al. (2022) Hypothyroidism confers tolerance to cerebral malaria. *Sci Adv*. 2022;8(14):eabj7110.
179. Yang J, et al. (2022) *Listeria monocytogenes* Inoculation Impedes the Development of Brain Pathology in Experimental Cerebral Malaria by Inhibition of Parasitemia. *ACS Infect Dis*. 2022;8(5):998-1009.
180. Bezerra Bellei JC, et al. (2022) A simple quinoline salt derivative is active in vitro against *Plasmodium falciparum* asexual blood stages and inhibits the development of cerebral malaria in murine model. *Chem Biol Interact*. 2022;355:109848.
181. Oliveira KRHM, et al. (2022) *Euterpe oleracea* fruit (Açai)-enriched diet suppresses the development of experimental cerebral malaria induced by *Plasmodium berghei* (ANKA) infection. *BMC Complement Med Ther*. 2022;22(1):11.
182. Wei W, et al. A Nanodrug Coated with Membrane from Brain Microvascular Endothelial Cells Protects against Experimental Cerebral Malaria. *Nano Lett*. 2022;22(1):211-219.
183. Kumar SP, Babu PP. (2022) NADPH Oxidase: a Possible Therapeutic Target for Cognitive Impairment in Experimental Cerebral Malaria. *Mol Neurobiol*. 2022;59(2):800-820.
184. Leleu I, et al (2022). A noncanonical autophagy is involved in the transfer of *Plasmodium*-microvesicles to astrocytes. *Autophagy*. 2022;18(7):1583-1598.
185. Akide Ndunge OB, et al. (2023) Effects of anti-tau immunotherapy on reactive microgliosis, cerebral endotheliopathy, and cognitive function in an experimental model of cerebral malaria. *J Neurochem*. 2023;167(3):441-460.
186. Plirat W, et al. (2023) Efficacy of artesunate combined with *Atractylodes lancea* or Prabchompoothaweep remedy extracts as adjunctive therapy for the treatment of cerebral malaria. *BMC Complement Med Ther*. 2023;23(1):332.
187. Jeje TO, et al. (2023) Antiplasmodial and interferon-gamma-modulating activities of the aqueous extract of stone breaker (*Phyllanthus niruri* Linn.) in malaria infection. *Parasitol Int*. 2023;97: 102789.
188. Nyariki JN, et al. Coenzyme Q10 exhibits anti-inflammatory and immune-modulatory thereby decelerating the occurrence of experimental cerebral malaria. *Mol Biochem Parasitol*. 2023;255: 111579.
189. Gul S, et al . Intravenous whole blood transfusion results in faster recovery of vascular integrity and increased survival in experimental cerebral malaria. *Mem Inst Oswaldo Cruz*. 2023;117:e220184.
190. Moreira ET, et al. (2024) Minocycline inhibits microglial activation in the CA1 hippocampal region and prevents long-term cognitive sequel after experimental cerebral malaria. *J Neuroimmunol*. 2024;397: 578480.
191. Silva TID, et al. (2024) Role of Nitric oxide synthase II in cognitive impairment due to experimental cerebral malaria. *Nitric Oxide*. 2024;153:41-49.
192. Jin Z, et al. (2024) Oral administration of IPI549 protects mice from neuropathology and an overwhelming inflammatory response during experimental cerebral malaria. *Int J Parasitol Drugs Drug Resist*. 2024;25:100539.
193. Shaham SH, et al. (2024) Role of angiotensin pathway and its target therapy to rescue from experimental cerebral malaria. *Microbes Infect*. 2024;26(4):105333.
194. Lv Y, et al. (2024) Extracellular vesicles derived from plasmodium-infected red blood cells alleviate cerebral malaria in *Plasmodium berghei* ANKA-infected C57BL/6J mice. *Int Immunopharmacol*. 2024;132:111982.
195. Silva AF, et al. (2024) Synthetic angiotensin II peptide derivatives confer protection against cerebral and severe non-cerebral malaria in murine models. *Sci Rep* **14**, 4682 .
196. Raza M, et al. (2024) Enhanced anti-malarial efficacy of mefloquine delivered via cationic liposome in a murine model of experimental cerebral malaria. *Eur J Pharm Biopharm*. 2024;197:114210.
197. Carpenter BA, et al. (2024) DHA-rich fish oil plays a protective role against experimental cerebral malaria by controlling inflammatory and mechanical events from infection. *J Nutr Biochem*. 2024;123:109492.

**Supplementary List of references by year and first author for the clinical trials on human cerebral malaria**

1. Rothe H. (1956) 100 cases of cerebral malaria. *East African Medical Journal* 1956;33(10):406–7
2. Kingston ME. (1971) Experience with urea in invert sugar for the treatment of cerebral malaria. *J Trop Med Hyg.* 1971;74(11):249-252.
3. Warrell DA, et al. (1982) Dexamethasone proves deleterious in cerebral malaria. A double-blind trial in 100 comatose patients. *N Engl J Med.* 1982;306(6):313-319.
4. Hoffman SL, et al. (1988) High-dose dexamethasone in quinine-treated patients with cerebral malaria: a double-blind, placebo-controlled trial. *J Infect Dis*. 1988;158(2):325-331.
5. White NJ, et al. (1988) Single dose phenobarbitone prevents convulsions in cerebral malaria. *Lancet*. 1988;2: 64–6.
6. Hemmer CJ, et al. (1991) Neither heparin nor acetylsalicylic acid influence the clinical course in human *Plasmodium falciparum* malaria: a prospective randomized study. *Am J Trop Med Hyg.* 1991;45: 608–612.
7. Gordeuk V, et al. (1992) Effect of iron chelation therapy on recovery from deep coma in children with cerebral malaria. *N Engl J Med.* 1992;327: 1473–1477.
8. Taylor TE, et al. (1992) Intravenous immunoglobulin in the treatment of paediatric cerebral malaria. *Clin Exp Immunol*. 1992;90: 357–362.
9. Krishna S, et al. (1994) Dichloroacetate for lactic acidosis in severe malaria: a pharmacokinetic and pharmacodynamic assessment. *Metabolism.* 1994;43:974–981.
10. Di Perri G, et al. (1995) Pentoxifylline as a supportive agent in the treatment of cerebral malaria in children. *J Infect Dis.* 1995; 171:1317–1322.
11. Krishna S, et al. (1995) Pharmacokinetics and pharmacodynamics of dichloroacetate in children with lactic acidosis due to severe malaria. *QJM.* 1995;88:341–349.
12. van Hensbroek MB, et al. (1996) The effect of a monoclonal antibody to tumor necrosis factor on survival from childhood cerebral malaria. *J Infect Dis.* 1996;174:1091–1097.
13. Krishna S, et al. (1996) The disposition and effects of two doses of dichloroacetate in adults with severe falciparum malaria. *Br J Clin Pharmacol.* 1996;41:29–34.
14. Hemmer CJ, et al. (1997) Supportive pentoxifylline in falciparum malaria: no effect on tumor necrosis factor alpha levels or clinical outcome: a prospective, randomized, placebo-controlled study. *Am J Trop Med Hyg.* 1997; 56:397–403.
15. Looareesuwan S, et al. (1998) Pentoxifylline as an ancillary treatment for severe falciparum malaria in Thailand. *Am J Trop Med Hyg.* 1998; 58:348–353.
16. Thuma PE, et al. (1998) Effect of iron chelation therapy on mortality in Zambian children with cerebral malaria. *Trans R Soc Trop Med Hyg.* 1998; 92:214–218.
17. Crawley J, et al. (2000) Effect of phenobarbital on seizure frequency and mortality in childhood cerebral malaria: a randomised, controlled intervention study. *Lancet.* 2000; 355:701–706.
18. Mohanty D, et al. (2002) Deferiprone (L1) as an adjuvant therapy for *Plasmodium falciparum* malaria. *Indian J Med Res.* 2002; 115:17–21.
19. Watt G, et al. (2002) A pilot study of N-acetylcysteine as adjunctive therapy for severe malaria. *QJM*. 2002; 95:285–290.
20. Das BK, et al. (2003) Pentoxifylline adjunct improves prognosis of human cerebral malaria in adults. *Trop Med Int Health.* 2003;8:680–684.
21. Treeprasertsuk S, et al. (2003) N-acetylcysteine in severe falciparum malaria in Thailand. *Southeast Asian J Trop Med Public Health*. 2003;34:37–42.
22. Agbenyega T, et al. (2003) Population kinetics, efficacy, and safety of dichloroacetate for lactic acidosis due to severe malaria in children. *J Clin Pharmacol.* 2003; 43:386–396.
23. Havlik I, et al. (2005) Curdlan sulphate in human severe/cerebral Plasmodium falciparum malaria. *Trans R Soc Trop Med Hyg.* 2005; 99:333–340.
24. Maitland K, et al. (2005) Randomized trial of volume expansion with albumin or saline in children with severe malaria: preliminary evidence of albumin benefit. *Clin Infect Dis.* 2005; 40:538–545.
25. Akech S, et al. (2006) Volume expansion with albumin compared to gelofusine in children with severe malaria: results of a controlled trial. *PLoS Clin Trials.* 2006; 1:e21.
26. Namutangula B, et al. (2007) Mannitol as adjunct therapy for childhood cerebral malaria in Uganda: a randomized clinical trial. *Malar J.* 2007; 6:138
27. Charunwatthana P, et al. (2009) N-acetylcysteine as adjunctive treatment in severe malaria: a randomized, double-blinded placebo-controlled clinical trial. *Crit Care Med.* 2009; 37:516–522.
28. Picot S, et al. (2009) Safety of epoietin beta-quinine drug combination in children with cerebral malaria in Mali. *Malar J.* 2009; 8:169.
29. Lell B, et al. (2010) Pentoxifylline as an adjunct therapy in children with cerebral malaria. *Malar J*. 2010; 9:368.
30. de Souza JB, et al. (2010) Oral activated charcoal prevents experimental cerebral malaria in mice and in a randomized controlled clinical trial in man did not interfere with the pharmacokinetics of parenteral artesunate. *PLoS One.* 2010; 5(4):e9867.
31. Mohanty S, et al. (2011) Brain swelling and mannitol therapy in adult cerebral malaria: a randomized trial. *Clin Infect Dis.* 2011; 53:349–355.
32. Maitland K, et al. (2011) Mortality after fluid bolus in African children with severe infection. *N Engl J Med.* 2011; 364:2483–2495.
33. Maude RJ, et al. (2011) Timing of enteral feeding in cerebral malaria in resource-poor settings: a randomized trial. *PLoS One.* 2011; 6(11):e27273.
34. Mwanga-Amumpaire J, et al. (2012) Effect of vitamin A adjunct therapy for cerebral malaria in children admitted to Mulago hospital: a randomized controlled trial. *Afr Health Sci*. 2012; 12(2):90-97.
35. Maude RJ, et al. (2014) Randomized controlled trial of levamisole hydrochloride as adjunctive therapy in severe falciparum malaria with high parasitemia. *J Infect Dis.* 2014; 209:120–129.
36. Hawkes MT, et al. (2015) Inhaled nitric oxide as adjunctive therapy for severe malaria: a randomized controlled trial. *Malar J.* 2015; 14:421.
37. Mwanga-Amumpaire J, et al. (2015) Inhaled nitric oxide as an adjunctive treatment for cerebral malaria in children: a Phase II randomized open-label clinical trial. *Open Forum Infect Dis.* 2015; 2:ofv111.
38. Cusick SE, et al. (2016) Comparison of iron status 28 d after provision of antimalarial treatment with iron therapy compared with antimalarial treatment alone in Ugandan children with severe malaria. *Am J Clin Nutr.* 2016;103(3):919-925.
39. Cusick SE, et al. (2020) Delayed iron improves iron status without altering malaria risk in severe malarial anemia. *Am J Clin Nutr.* 2020; 111(5):1059-1067.
40. Ssemata AS, et al. (2020) Delayed iron does not alter cognition or behavior among children with severe malaria and iron deficiency. *Pediatr Res.* 2020; 88(3):429-437.
41. Birbeck GL, et al. (2024) Acetaminophen and Ibuprofen in Pediatric Central Nervous System Malaria: A Randomized Clinical Trial. *JAMA Neurol*. 2024;81(8):857-865.
