# Supplementary material for: Cerebral malaria: of mice and men
Source: Trans R Soc Trop Med Hyg. 2025 Nov 14;120(3):254–7. doi: 10.1093/trstmh/traf126 (PMC13017480; doi:10.1093/trstmh/traf126)
Supplement: traf126_Supplemental_Files [file traf126_supplemental_files.zip › Table S1.docx]

**Table S1.**

**Full referenced list of interventions evaluated in the murine model of cerebral malaria from 1987 to 2024.**

|  | **Intervention** | **Effect** | **Parasite** | **Mice** | **Authors** |
| --- | --- | --- | --- | --- | --- |
| [S1] | Anti-TNF antibody | Reduced CM Improved survival | *Plasmodium berghei* ANKA | CBA/Ca | Grau *et al.,* 1987 |
| [S2] | Cyclosporine | Reduced CM Improved survival | *Plasmodium berghei* ANKA | CBA/Ca | Grau *et al.,* 1988 |
| [S3] | anti-rGM-CSF and anti-rIL-3 antibodies | Reduced CM | *Plasmodium berghei* ANKA | CBA | Grau *et al.,* 1988 |
| [S4] | Antioxidants | Reduced CM | *Plasmodium berghei* ANKA | A/J and CBA/H | Thurmwood *et al.,* 1989 |
| [S5] | Anti gamma interferon antibody | Reduced CM Improved survival | *Plasmodium berghei* ANKA | CBA/Ca | Grau *et al.,* 1989 |
| [S6] | IL-1 | Reduced CM Improved survival | *Plasmodium berghei* K 173 | C57Bl/6J | Curfs *et al.,* 1990 |
| [S7] | Anti LFA1 (CD11a) antibody | Reduced CM Improved survival | *Plasmodium berghei* ANKA | CBA/Ca | Grau *et al.,* 1991 |
| [S8] | Pentoxifylline | Reduced CM Improved survival | *Plasmodium berghei* ANKA | CBA/Ca | Kremsner *et al.,* 1991 |
| [S9] | Iloprost | Reduced CM Improved survival | *Plasmodium berghei* ANKA | CBA/Ca | Sliwa *et al.,* 1991 |
| [S10] | Fish Oil | Reduced CM Improved survival | *Plasmodium berghei* ANKA | C57B1/6 | Blok *et al.,* 1992 |
| [S11] | Indomethacin | No effect | *Plasmodium berghei* ANKA | C57B1/6 | Blok *et al.,* 1992 |
| [S12] | Starvation | Prevention of CM | *Plasmodium berghei* ANKA | CBA/T6 | Hunt *et al.,* 1993 |
| [S13] | Murine AIDS | Prevention of CM | *Plasmodium berghei* ANKA | C57/BL/6 | Eckwalanga *et al.,* 1995 |
| [S14] | Fish oil | Prevention of CM | *Plasmodium berghei* ANKA | CBA/CaJ | Levander *et al.,* 1995 |
| [S15] | Dexamethasone | Reduced CM Improved survival | *Plasmodium berghei* ANKA | CBA/T6 and DBA/2J | Neill & Hunt 1995 |
| [S16] | Fatty acids | Reduced CM | *Plasmodium berghei* ANKA | C57/BL/6 | Moumaris *et al.,* 1995 |
| [S17] | Coinfection with *B.pahangi* | Reduced CM Improved survival | *Plasmodium berghei* | CBA/J | Yan *et al.,* 1997 |
| [S18] | CD4 or CD8 depletion | Reduced CM | *Plasmodium berghei* K 173 | C57B1/6J and C57B1/10 | Hermsen *et al.,* 1997 |
| [S19] | Recombinant IL-10 | Reduced CM | *Plasmodium berghei* ANKA | CBA/J | Kossodo *et al.,* 1997 |
| [S20] | Splenectomy | Reduced CM Improved survival | *Plasmodium berghei* K 173 | C57BL/6J | Hermsen *et al.,* 1998 |
| [S21] | Thiolated recombinant TNF (rhTNF alpha-AT) | Reduced CM Improved survival | *Plasmodium berghei* K173 | C57BL/6J | Postma *et al.,* 1999 |
| [S22] | Liposomal recombinant TNF | Reduced CM Improved survival | *Plasmodium berghei* K173 | C57BL/6J | Postma *et al.,* 1999 |
| [S23] | Dichloroacetate | Improved survival | *Plasmodium berghei* ANKA | CBA/T6 | Rae *et al.,* 2000 |
| [S24] | Immunisation with synthetic GPI | Prevents CM and death | *Plasmodium berghei* ANKA | C57BL6 | Schofield *et al.,* 2002 |
| [S25] | Anti CD 41 | Reduced CM Improved survival | *Plasmodium berghei* ANKA | C57BL/6 | Sun *et al.,* 2003 |
| [S26] | Anti CD 61 | Reduced CM Improved survival | *Plasmodium berghei* ANKA | C57BL/6 | Sun *et al.,* 2003 |
| [S27] | Celecoxib | Earlier onset of CM | *Plasmodium berghei* ANKA | CBA or C57BL6 | Ball *et al.,* 2004 |
| [S28] | kynurenine-3-hydroxylase inhibitor | Reduced CM Improved survival | *Plasmodium berghei* ANKA | C57BL/6J | Clark *et al.,* 2005 |
| [S29] | Thalidomide | Improved survival | *Plasmodium berghei* ANKA | CBA | Muniz-Junqueira *et al.,* 2005 |
| [S30] | Erythropoietin | Improved survival | *Plasmodium berghei* ANKA | CBA/J | Kaiser *et al.,* 2006 |
| [S31] | Nitric oxide | Reduced CM Improved survival | *Plasmodium berghei* ANKA | C57BL/6 | Gramaglia *et al.,* 2006 |
| [S32] | anti-BTLA mAb | Reduced CM | *Plasmodium berghei* ANKA | C57BL/6 | Lepenies *et al.,* 2007 |
| [S33] | Carbon monoxide | Reduced CM Improved survival | *Plasmodium berghei* ANKA | C57BL/6 | Pamplona *et al.,* 2007 |
| [S34] | Recombinant human IFN-alpha | Reduced CM Improved survival | *Plasmodium berghei* ANKA | C57BL/6 | Vigario *et al.,* 2007 |
| [S35] | anti CD25 antibody | Reduced CM Improved survival | *Plasmodium berghei* ANKA | CBA and C57BL/6 | Amante *et al.,* 2007 |
| [S36] | CD8 T cell depletion | Prevention or attenuation of CM | *Plasmodium berghei* ANKA | CBA and C57BL/6 | Randall *et al.,* 2008 |
| [S37] | Guanylhydrazone CNI-1493 | Reduced CM Improved survival | *Plasmodium berghei* ANKA | C57BL/6 | Specht *et al.,* 2008 |
| [S38] | Antibody blockade of C5a or C5a receptor (C5aR) | Reduced CM Improved survival | *Plasmodium berghei* ANKA | C57BL/6 | Patel *et al.,* 2008 |
| [S39] | Pantethine | Reduced CM Improved survival | *Plasmodium berghei* ANKA | CBA/J | Penet *et al.,* 2008 |
| [S40] | Simvastatin | No effect | *Plasmodium berghei* ANKA | C57BL/6 | Kobbe *et al.,* 2008 |
| [S41] | Aspirin | Reduced CM Improved survival | *Plasmodium berghei* ANKA | C57BL/6 | Srivastava *et al.,* 2008 |
| [S42] | Clopidogrel | Reduced CM Improved survival | *Plasmodium berghei* ANKA | C57BL/6 | Srivastava *et al.,* 2008 |
| [S43] | Anti-apoptotic strategies | No effect | *Plasmodium berghei* ANKA | C57BL/6 | Helmers *et al.,* 2008 |
| [S44] | Obesity | Prevented CM Improved survival | *Plasmodium berghei* ANKA | C57BL/6 | Robert *et al.,* 2008 |
| [S45] | Hyperbaric oxygen | Prevented CM Improved survival | *Plasmodium berghei* ANKA | C57BL/6 | Blanco *et al.,* 2008 |
| [S46] | Glatiramer | Reduced CM Improved survival | *Plasmodium berghei* ANKA | C57BL/6 | Lackmer *et al.,* 2009 |
| [S47] | IP 10 blockade | Reduced CM Improved survival | *Plasmodium berghei* ANKA | C57BL/6 | Nie *et al.,* 2009 |
| [S48] | Rosiglitazone | Reduced CM Improved survival | *Plasmodium berghei* ANKA | C57BL/6 | Serghides *et al.*, 2009 |
| [S49]* | Artemisone together with chloroquine | Complete cure | *Plasmodium berghei* ANKA | C57BL/6 | Waknine-Grinberg *et al.,* 2010 |
| [S50] | Rodent filarial infection by *Litomosoides sigmodontis* | Reduced inflammation | *Plasmodium berghei ANKA* | C57BL/6 | Specht *et al.,*2010 |
| [S51] | Fasudil | Prevented CM | *Plasmodium berghei* ANKA | *ICR* | Waknine-Grinberg *et al.,*2010 |
| [S52] | *S.mansoni* infection | Reduced CM Improved survival | *Plasmodium berghei* ANKA | C57BL/6 | Waknine-Grinberg *et al.,*2010 |
| [S53] | Vitamin E | No improvement in symptoms, parasitaemia and survival | *Plasmodium berghei* ANKA | C57BL/6 | Herbas *et al.,* 2010 |
| [S54] | Nimodipine | Increased survival | *Plasmodium berghei* ANKA | C57BL/6 | Cabrales *et al.,* 2010 |
| [S55] | Vitamin A + DTP | Increased parasitaemia Increased CM | *Plasmodium berghei* ANKA | C57BL/6 | Hein-Kristensen *et al.,*2010 |
| [S56] | Oral activated charcoal | Prevented CM | *Plasmodium berghei* ANKA | C57BL/6 | de Souza *et al.,* 2010 |
| [S57] | IL-2/anti-IL-2 complexes | Prevented CM | *Plasmodium berghei* ANKA | C57BL/6 | Haque *et al.,*2010 |
| [S58] | Inhaled nitric oxide | Improved CM  Reduced parasitaemia Improved survival | *Plasmodium berghei* ANKA | C57BL/6 | Serghides *et al.,*2011 |
| [S59] | lentiviral vector-mediated overexpression of hepcidin (pLenti-Hep-EGFP) | Reduced parasitaemia Improved survival | *Plasmodium berghei* ANKA | ICR and Kunming strain | Wang *et al.,*2011 |
| [S60] | Sphingosine 1- pathway modulators: FTY720, LX2931 | Improved survival | *Plasmodium berghei* ANKA | C57BL/6 | Finney *et al.,*2011 |
| [S61] | NO donor (dipropylenetriamine NONOate [DPTA-NO]) | Prevented CM | *Plasmodium berghei* ANKA | C57BL/6 | Cabrales *et al.,* 2011 |
| [S62] | Amodiaquine-derived NO-donor, | Prolonged survival | *Plasmodium berghei* ANKA | C57BL/6 | Bertinaria *et al.,*2011 |
| [S63] | TLR7 and TLR9 antagonist, E6446 | Prevented complications/ inflammation | *Plasmodium berghei* ANKA | C57BL/6 | Franklin *et al.,*2011 |
| [S64] | Beta interferon | Reduced inflammation Improved CM | *Plasmodium berghei* ANKA | C57BL/6 | Morrell *et al.,* 2011 |
| [S65]* | Artemether | Cured CM | *Plasmodium berghei* ANKA | C57BL/6 | Clemmer *et al.,* 2011 |
| [S66] | Attenuated Plasmodium *berghei* parasites | Reduced parasitaemia Prevented CM | *Plasmodium berghei* ANKA | C57BL/6 | Gerald *et al.,* 2011 |
| [S67] | FMS-like tyrosine kinase 3 (FLT3) ligand | Prevented CM | *Plasmodium berghei* ANKA | C57BL/6 | Tamura *et al.,* 2011 |
| [S68] | S-nitrosoglutathione, an NO donor | Prevented CM | *Plasmodium berghei* ANKA | C57BL/6 | Zanini *et al.,* 2011 |
| [S69] | Lovastatin | Prevented inflammation and CM | *Plasmodium berghei* ANKA | C57BL/6 | Reis *et al.,*2012 |
| [S70] | Lithium | Reduced neurocognitive impairment | *Plasmodium berghei* ANKA | C57BL/6 | Dai *et al.,* 2012 |
| [S71] | ETA receptor antagonist HJP-272 | Improved survival | *Plasmodium berghei* ANKA | C57BL/6 | Dai *et al.,* 2012 |
| [S72] | Erythropoietin | Reduced severity of CM Improved survival | *Plasmodium berghei* ANKA | C57BL/6 | Hempel *et al.,* 2012 |
| [S73]* | Chloroquine | Prevented CM | *Plasmodium berghei* ANKA | C57BL/6 | Zhu *et al.,* 2012 |
| [S74] | S-nitrosoglutathione (GSNO) | Reduced parasitaemia Prevented CM | *Plasmodium berghei* ANKA | C57BL/6 | Zanini *et al.,* 2012 |
| [S75] | sildenafil plus DPTA-NO | Reduced ECM | *Plasmodium berghei* ANKA | C57BL/6 | Martins *et al.,* 2012 |
| [S76] | Atorvastatin | Improved survival Reduced parasitaemia Prevented CM | *Plasmodium berghei* ANKA | CBA/J | Souraud *et al.,* 2012 |
| [S77] | Carbon monoxide-releasing molecule (CO-RM; ALF492) | Prevented CM | *Plasmodium berghei* ANKA | C57BL/6 | Pena *et al.,* 2012 |
| [S78]* | Artemiside | Prolonged survival Reduced parasitaemia | *Plasmodium berghei* ANKA | C57BL/6 | Guo *et al.,* 2012 |
| [S79]* | Artesunate | Reduced inflammation Reduced cognitive impairment Improved survival | *Plasmodium berghei* ANKA | C57BL/6 | Miranda *et al.,* 2013 |
| [S80] | Glucocorticosteroids in nano-sterically stabilized liposomes | Complete cure Reduced inflammation Reduced sequelae | *Plasmodium berghei* ANKA | C57BL/6 and ICR | Waknine-Grinberg *et al.,* 2013 |
| [S81] | Atorvastatin | Reduced CM Improved survival Reduced parasitaemia | *Plasmodium berghei* ANKA | C57BL/6 | Dormoi *et al.,* 2013 |
| [S82] | *Azadirachta indica* ethanolic extract | Prevented complications | *Plasmodium berghei* ANKA | Swiss albino mice | Bedri *et al.,* 2013 |
| [S83] | Transdermal glyceryl trinitrate | Increased survival Reduced complications | *Plasmodium berghei* ANKA | C57BL/6 | Orjuela-Sánchez *et al.,* 2013 |
| [S84] | Atorvastatin | Reduced CM improved survival | *Plasmodium berghei* ANKA | C57BL/6 | Wilson *et al.,* 2013 |
| [S85] | Nimodipine | Increased survival Improved CM | *Plasmodium berghei* ANKA | C57BL/6 | Martins *et al.,*2013 |
| [S86]* | Methylene blue | Improved CM Improved survival Reduced parasitaemia | *Plasmodium berghei* ANKA | C57BL/6 | Dormoi *et al.,* 2013 |
| [S87] | Methylene blue and atorvastatin | Improved survival | *Plasmodium berghei* ANKA | C57BL/6 | Dormoiivar *et al.,*2013 |
| [S88] | Hydrogen sulfide gas | Not effective | *Plasmodium berghei* ANKA | C57BL/6 | DellaValle *et al.,*2013 |
| [S89] | Reversible JNK pathway inhibitor SP600125 | Improved survival Reduced neuronal cell death | *Plasmodium berghei* ANKA | C57BL/6 | Anand *et al.,*2013 |
| [S90] | rh-Erythropoietin | Improved CM | *Plasmodium berghei* ANKA | C57BL/6 | Karlsson *et al.,* 2013 |
| [S91] | Erythropoietin | Improved survival | *Plasmodium berghei* ANKA | C57BL/6 | Wei *et al.,*2013 |
| [S92] | Lipoxin A₄ and 15-epi-lipoxin A₄ | Prolonged survival | *Plasmodium berghei* ANKA | C57BL/6 | Shryock *et al.,* 2013 |
| [S93]* | Intranasal artesunate | Improved survival Reduced parasitaemia | *Plasmodium berghei* ANKA | CBA/J mice | Marijon *et al.,* 2014 |
| [S94] | Lambda-carrageenan | Increased severity  Increased mortality | *Plasmodium berghei* ANKA | BALB/c mice | Recuenco *et al.,* 2014 |
| [S95] | Liposome-encapsulated betamethasone hemisuccinate (nSSL-BMS) | Prevented CM Increased survival | *Plasmodium berghei* ANKA | C57BL/6 | Guoalry *et al.,* 2014 |
| [S96] | Vitamin D | Improved CM | *Plasmodium berghei* ANKA | C57BL/6 | He *et al.,* 2014 |
| [S97]* | Artemisone in combination with conventional antimalarial drugs | Prevented CM Reduced parasitaemia | *Plasmodium berghei* ANKA | C57BL/6 | Guiguemde *et al.,* 2014 |
| [S98] | PPARγ agonists | Reduced neurocognitive impairment | *Plasmodium berghei* ANKA | C57BL/6 | Serghides *et al.,* 2014 |
| [S99] | Tempol | Partial protection against CM | *Plasmodium berghei* ANKA | C57BL/6 | Francischetti *et al.,* 2014 |
| [S100] | Neuregulin-1 | Increased parasites. Improved survival | *Plasmodium berghei* ANKA | C57BL/6 | Solomon *et al.,* 2014 |
| [S101] | Citicoline | Reduced parasitaemia Increased survival prevent CM | *Plasmodium berghei* ANKA | CBA | El-Assaad *et al.,* 2014 |
| [S102] | 6-diazo-5-oxo-L-norleucine (DON) | Improved CM | *Plasmodium berghei* ANKA | C57BL/6 | Gordon *et al.,* 2015 |
| [S103] | Vascular endothelial growth factor (VEGF) and lovastatin | Prevented CM | *Plasmodium berghei* ANKA | C57BL/6 | Canavese *et al.,*2015 |
| [S104] | VEGF and LPS | Prevented CM Improved survival | *Plasmodium berghei* ANKA | C57BL/6, Balb/c | Canavese *et al.,* 2015 |
| [S105] | Angiotensin -2 | Prevented CM Increased survival | *Plasmodium berghei* ANKA | C57BL/6 | Gallego-Delgado *et al.,* 2015 |
| [S106] | NO-Donor | Increased survival | *Plasmodium berghei* ANKA | C57BL/6 | Bertinaria *et al.,* 2015 |
| [S107] | Proteasome inhibitors - bortezomib | No improvement in survival | *Plasmodium berghei* ANKA | C57BL/6 | Howland *et al.,* 2015 |
| [S108] | Formulated diet to control iron status | Improved survival | *Plasmodium berghei* ANKA | H67D | Leitner *et al.,* 2015 |
| [S109] | Curcumin | Reduced inflammation | *Plasmodium berghei* ANKA | C57BL/6 | Dende *et al.,* 2015 |
| [S110] | Rapamycin inhibitor | Prevented CM | *Plasmodium berghei* ANKA | C57BL/6 | Gordon *et al.,* 2015 |
| [S111] | RRx-001  Anticancer drug | Improved inflammation/CM | *Plasmodium berghei* ANKA | C57BL/6 | Yalcin *et al.,* 2015 |
| [S112] | Mesenchymal stromal cell therapy | Increased survival Reduced parasitaemia | *Plasmodium berghei* ANKA | C57BL/6 | Souza *et al.,* 2015 |
| [S113] | L-arginine | Reduced survival  Increased inflammation | *Plasmodium berghei* ANKA | C57BL/6 | Xu *et al.,* 2015 |
| [S114] | Iron dextran | Prevented CM | *Plasmodium berghei* ANKA | C57BL/6 | Van *et al.,* 2015 |
| [S115] | Dietary restriction | Reduced parasite accumulation in brain | *Plasmodium berghei* ANKA | C57BL/6 | Mejia *et al.,* 2015 |
| [S116] | Cannabidiol | Increased survival  Rescue of cognitive function | *Plasmodium berghei* ANKA | C57BL/6 | Campos *et al.,* 2015 |
| [S117] | *Agaricus blazei* extract | Reduced CM Reduced parasitaemia Increased survival Reduced symptoms | *Plasmodium berghei* ANKA | C57BL/6 | Val *et al.,* 2015 |
| [S118] | High dietary folate | Higher parasitaemia  Reduced survival | *Plasmodium berghei* ANKA | C57BL/6 | Meadows *et al.,* 2015 |
| [S119]* | Nanostructured lipid carriers of artemether-lumefantrine | Improved CM | *Plasmodium berghei* ANKA | C57BL/6 | Prabhu *et al.,* 2016 |
| [S120] | Intramuscular vitamin D | Cured CM | *Plasmodium berghei* ANKA | C57BL/6 | Dwivedi *et al.,* 2016 |
| [S121] | Cysteamine | Reduced parasitaemia Prevented CM | *Plasmodium berghei* ANKA | A/J mice | Moradin *et al.,* 2016 |
| [S122] | Endothelin-1 | No effect on parasitaemia Increased severity of CM | *Plasmodium berghei* ANKA | C57BL/6 | Martins *et al.,* 2016 |
| [S123] | Synthetic oleanane triterpenoids | Improved survival Improved CM | *Plasmodium berghei* ANKA | C57BL/6 | Crowley *et al.,* 2017 |
| [S124] | Rapamycin | Improved survival Improved CM | *Plasmodium berghei* ANKA | C57BL/6 | Mejia *et al.,* 2017 |
| [S125] | Nanocurcumin | Inhibited sequestration | *Plasmodium berghei* ANKA | C57BL/6 | Dende *et al.,* 2017 |
| [S126] | Protein Tyrosine Phosphatase Inhibition | Prevented CM | *Plasmodium berghei* ANKA | C57BL/6 | van den Ham *et al.,* 2017 |
| [S127] | MEK1/2 inhibitor | Reduced parasitaemia Improved CM | *Plasmodium berghei* ANKA | C57BL/6 | Wu *et al.,* 2017 |
| [S128]* | Trioxaquine | Reduced parasitaemia Improved survival | *Plasmodium berghei* ANKA | C57BL/6 | Odhiambo *et al.,* 2017 |
| [S129] | Hydrogen sulfide | Improved CM | *Plasmodium berghei* ANKA | C57BL/6 | Jiang *et al.,* 2017 |
| [S130] | Erythropoietin | Improved CM Improved survival | *Plasmodium berghei* ANKA | C57BL/6 | Du *et al.,* 2017 |
| [S131] | *Zizyphus spina* extract (ZLE) | Improved CM | *Plasmodium berghei* ANKA | C57BL/6 | Mubarakid *et al.,* 2017 |
| [S132]* | Controlled release artemisone | Prevented CM | *Plasmodium berghei* ANKA | C57BL/6 | Golenser *et al.,* 2017 |
| [S133] | Trappin-2/elafin | Reduced parasitaemia | *Plasmodium berghei* ANKA | C57BL/6 | Roussilhon *et al.,* 2017 |
| [S134]* | Minocycline | Improved survival Prevented sequelae Reduced parasitaemia | *Plasmodium berghei* ANKA | C57BL/6 | Apoorv *et al.,*  2017 |
| [S135] | Tacrolimus | Prevented CM | *Plasmodium berghei* ANKA | C57BL/6 | Bao *et al.,* 2017 |
| [S136] | Glutamate receptor antagonist MK801 | Prevented sequelae | *Plasmodium berghei* ANKA | C57BL/6 | de Miranda *et al.,* 2017 |
| [S137] | Glutaminase inhibitor JHU-083 | Improved CM Improved survival | *Plasmodium berghei* ANKA | C57BL/6 | Riggle *et al.,* 2018 |
| [S138] | L-arginine | Improved survival | *Plasmodium berghei* ANKA | C57BL/6 | Ong *et al.,* 2018 |
| [S139] | Vitamin D | Improved CM Improved survival | *Plasmodium berghei* ANKA | C57BL/6 | Wu *et al.,* 2018 |
| [S140]* | Artesunate combination therapy | Reduced parasitaemia Improved CM Increased survival | *Plasmodium berghei* ANKA | C57BL/6 | Jiang *et al.,* 2018 |
| [S141] | α-Tocopheryl succinate | Reduced parasitaemia Increased survival | *P. yoelii* 17XL and *P. berghei* ANKA | C57BL/6 mice | Kume *et al.,* 2018 |
| [S142] | IL-33 | Improved CM Improved survival | *Plasmodium berghei* ANKA | C57BL/6 mice | Strangward *et al.,* 2018 |
| [S143] | Neuregulin-1 | Improved survival | *Plasmodium berghei* ANKA | C57BL/6 mice | Liu *et al.,* 2018 |
| [S144] | Interleukin-15 Complex Treatment | Improved survival | *Plasmodium berghei* ANKA | C57BL/6 mice | Burrack *et al.,* 2018 |
| [S145] | Rocaglate | Reduced parasitaemia Improve CM | *Plasmodium berghei* ANKA | C57BL/6 mice | Langlais *et al.,* 2018 |
| [S146] | Single intravenous or subcutaneous whole-parasite immunization | Delayed parasitaemia | *Plasmodium berghei* ANKA | C57BL/6 mice | Heiss *et al.,* 2018 |
| [S147]* | Artemether in nanostructured lipid carrier | Prevented recrudescence | *Plasmodium berghei* ANKA | C57BL/6 mice | Vanka *et al.,*2018 |
| [S148] | Doxycycline* | Prevented CM | *Plasmodium berghei* ANKA | C57BL/6 mice | Schmidt *et al.,* 2018 |
| [S149] | Ethanolic extract *Trichoderma stromaticum* | Improved survival prevented sequelae | *Plasmodium berghei* ANKA | C57BL/6 mice | Cariaco *et al.,* 2018 |
| [S150] | Concurrent Chikungunya infection | Improved survival Improved CM | *Plasmodium berghei* ANKA | C57BL/6 mice | Teo *et al.,*2018 |
| [S151] | Perillyl alcohol | Reduced inflammation | *Plasmodium berghei* ANKA | C57BL/6 mice | Rodriguez *et al.,* 2018 |
| [S152] | *Terminalia albida* | Reduced inflammation Reduced parasitaemia | *Plasmodium berghei* ANKA | C57BL/6 mice | Camara *et al.,*2019 |
| [S153] | Fenozyme | Increased survival Improved CM | *Plasmodium berghei* ANKA | C57BL/6 mice | Zhao *et al.,*2019 |
| [S154] | L-arginine supplementation and thromboxane synthase inhibition | Improved CM | *Plasmodium berghei* ANKA | C57BL/6 mice | Moreira *et al.,* 2019 |
| [S155] | T-cell Immunomodulatory Protein | Reduced pathology | *Plasmodium berghei* ANKA | C57BL/6 and BALB/c mice | Cui *et al.,* 2019 |
| [S156] | Coenzyme Q10 | Improved survival Improved CM Reduced inflammation | *Plasmodium berghei* ANKA | C57BL/6 | Nyariki *et al.,* 2019 |
| [S157] | Citrulline | Improved survival | *Plasmodium berghei* ANKA | C57BL/6 | Gramaglia *et al.,* 2019 |
| [S158] | PDL1 Fusion Protein | Improved survival | *Plasmodium berghei* ANKA | C57BL/6 | Wang *et al.,* 2019 |
| [S159] | Melatonin | Improved CM Reduced complications | *Plasmodium berghei* ANKA | Swiss albino mice | Ataide *et al.,* 2020 |
| [S160] | Nanotized curcumin-benzothiophene conjugate | Reduced parasitaemia Improved survival | *Plasmodium berghei* ANKA | C57BL/6 | Ghosh *et al.,* 2020 |
| [S161]* | Benzene Aminoquinolines | Reduced parasitaemia Improved survival | *Plasmodium berghei* ANKA | C57BL/6 | Srbljanović *et al.,* 2020 |
| [S162] | PD1 fusion protein | Prevented neurological sequelae | *Plasmodium berghei* ANKA | C57BL/6 | Jiang *et al.,* 2020 |
| [S163] | Mesenchymal stromal cells | Improved CM Reduced sequelae | *Plasmodium berghei* ANKA | C57BL/6 | Lima *et al.,* 2020 |
| [S164] | Tetramethylpyrazine | Improved CM | *Plasmodium berghei* ANKA | C57BL/6 | Zheng *et al.,* 2021 |
| [S165]* | Artemisone microemulsion (ME) | Prevented CM | *Plasmodium berghei* ANKA | C57BL/6 | Zech *et al.,* 2021 |
| [S166] | Whole blood transfusion | Improved survival | *Plasmodium berghei* ANKA | C57BL/6 | Gul *et al.,* 2021 |
| [S167] | Monocyte Locomotion Inhibitory Factor (MLIF) | Increased survival. Prevented neurological sequelae | *Plasmodium berghei* ANKA | C57BL/6 | Galán-Salinas *et al.,* 2021 |
| [S168]* | Artesunate-loaded nanostructured lipid carriers for intranasal administration | Reduced parasitaemia | *Plasmodium berghei* ANKA | CBA/J | Agbo *et al.,* 2021 |
| [S169] | Anti-CD47 | Reduced inflammation; Improved CM Improved survival | *Plasmodium berghei* ANKA | C57BL/6 | Torrez *et al.,* 2021 |
| [S170]* | Self-microemulsifying drug delivery system (SMEDDS) for artemisone | Improved CM | *Plasmodium berghei* ANKA | C57BL/6 | Zech *et al.,* 2021 |
| [S171] | IL-4 | Reduced parasitaemia Improved CM Increased survival | *Plasmodium berghei* ANKA | C57BL/6 | Wu *et al.,* 2021 |
| [S172] | Anti-CD146 antibody AA98 | Improved CM Increased survival | *Plasmodium berghei* ANKA | C57BL/6 | Duan *et al.,* 2021 |
| [S173] | Anti-Lt-α antibody | Increased survival Improved CM | *Plasmodium berghei* ANKA | C57BL/6 | Eeka *et al.,* 2022 |
| [S174] | α-Tocopheryloxy Acetic Acid | Improved CM | *Plasmodium berghei* ANKA | C57BL/6 | Ariefta *et al.,* 2022 |
| [S175] | Griseofulvin | Improved and prevented CM | *Plasmodium berghei* ANKA | C57BL/6 | Chandana *et al.,* 2022 |
| [S176] | Glucose-modified nanocomposite liposomes | Reduced infection rate and recurrence | *Plasmodium berghei* ANKA | C57BL/6 | Tian *et al.,* 2022 |
| [S177] | Monoterpene 1,8-cineole | Improved CM Reduced parasitaemia | *Plasmodium berghei* ANKA | C57BL/6 | Santos *et al.,* 2022 |
| [S178] | Hypothyroidism, Sirtuin 1 | Improved CM Reduced mortality | *Plasmodium berghei* ANKA | C57BL/6 | Rodriguez-Muñoz *et al.,* 2022 |
| [S179] | *Listeria monocytogenes* Inoculation | Improved CM | *Plasmodium berghei* ANKA | C57BL/6 | Yang *et al.,* 2022 |
| [S180]* | Quinoline salt derivative | Reduced parasitaemia Prevented CM | *Plasmodium falciparum* | C57BL/6 | Bezerra Bellei *et al.,* 2022 |
| [S181] | *Euterpe oleracea* fruit (Açai)-enriched diet | Improved survival Improved CM Reduced neurocognitive deficits | *Plasmodium berghei* ANKA | Swiss albino mice | Oliveira *et al.,* 2022 |
| [S182]* | Nanoparticle formulation dihydroartemisinin coated in a brain microvascular endothelial cell derived biomimetic membrane | Improved CM Improved survival | *Could not be found as publication could not be accessed | *Could not be found | Wei *et al.,* 2022 |
| [S183] | Apocynin | Improved CM Reduced long-term effects | *Plasmodium berghei* ANKA | C57BL/6 | Kumar *et al.,* 2022 |
| [S184] | 3-methyladenine bafilomycin A1 | Improved CM | *Plasmodium berghei* ANKA | C57BL/6 | Leleu *et al.,* 2022 |
| [S185] | Monoclonal antibody to pathological tau (PHF-1 mAB) | Reduced inflammation Reduced cognitive impairment | *Plasmodium berghei* ANKA | C57BL/6 | Akide Ndunge *et al.,* 2023 |
| [S186] | *Atractylodes lancea* or *Prabchompoothawe*sp remedy extracts | Combination: Improved CM Increased survival Reduced cognitive impairment | *Plasmodium berghei* ANKA | C57BL/6 mice | Plirat *et al.,* 2023 |
| [S187] | aqueous extract *Phyllanthus niruri* | Reduced parasitaemia Increased survival Reduced inflammation | *Plasmodium berghei* ANKA | C57BL/6 mice | Jeje *et al.,* 2023 |
| [S188] | Coenzyme Q10 | Reduced parasitaemia, Improved survival | *Plasmodium berghei* ANKA | C57BL6 | Nyakiri *et al.,* 2023 |
| [S189] | Whole blood transfusion | Increased survival Improved CM | *Plasmodium berghei* ANKA | C57BL6 | Gul *et al.,* 2023 |
| [S190]* | Minocycline | Reduced proinflammatory cytokines Prevented long-term cognitive decline | *Plasmodium berghei* ANKA | C57BL6 | Moreira *et al.,* 2024 |
| [S191] | Aminoguanidine | Reduced severity of CM Prevented long-term sequelae | *Plasmodium berghei* ANKA | C57BL6 | Silva *et al.,* 2024 |
| [S192] | IPI549 | Reduced inflammation Prevented CM | *Plasmodium berghei* ANKA | C57BL/6J | Jin *et al.,* 2024 |
| [S193] | Irbesartan and losartan | Improved survival Reduced inflammation | *Plasmodium berghei* ANKA | C57BL6 mice | Shaham *et al.,* 2024 |
| [S194] | Extracellular vesicle immunization | Improved survival | *Plasmodium berghei* ANKA | C57BL/6J | Lv *et al.,* 2024 |
| [S195] | Angiotensin-2 derivatives | Reduced parasitaemia Increased survival | *Plasmodium berghei* ANKA | BALB/c mice | Silva *et al.,* 2024 |
| [S196]* | Liposomal mefloquine | Improved CM | *Plasmodium berghei* ANKA | *Could not be found as publication could not be accessed | Raza *et al.,* 2024 |
| [S197] | DHA-rich fish oil | Improved CM Improved survival | *Plasmodium berghei ANKA* | C57BL/6 | Carpenter *et al.,* 2024 |

*antimalarial drugs; expected to be successful in comparison with no treatment
