# Supplementary material for: Cerebral malaria: of mice and men
Source: Trans R Soc Trop Med Hyg. 2025 Nov 14;120(3):254–7. doi: 10.1093/trstmh/traf126 (PMC13017480; doi:10.1093/trstmh/traf126)
Supplement: traf126_Supplemental_Files [file traf126_supplemental_files.zip › Table S2.docx]

**Table S2**

List of clinical trials on human cerebral malaria from 1956 to 2024 – includes clinical trials on severe malaria that included patients with cerebral malaria

| Number | Intervention | Effect | Reference | Year |
| --- | --- | --- | --- | --- |
| [S198] | Urea | No benefit | Rothe 1956 | 1956 |
| [S199] | Urea | No benefit | Kingston 1971 | 1971 |
| [S200] | Dexamethasone | Harmful | Warrell *et al.,* 1982 | 1982 |
| [S201] | High dose Dexamethasone | No benefit | Hoffman *et al.,* 1988 | 1988 |
| [S202] | Low dose Phenobarbital | Fewer convulsions | White *et al.,* 1988 | 1988 |
| [S203] | Heparin or acetylsalicilic acid | No benefit | Hemmer *et al.,* 1991 | 1991 |
| [S204] | Deferoxamine | No benefit | Gordeauk *et al.,* 1992 | 1992 |
| [S205] | Immunoglobulin | Harmful | Taylor *et al.,* 1992 | 1992 |
| [S206] | Dichloroacetate | No benefit | Krishna *et al.,* 1994 | 1994 |
| [S207] | Pentoxyfylline | No benefit | Di Perri *et al.,* 1995 | 1995 |
| [S208] | Dichloroacetate | No benefit | Krishna *et al.,* 1995 | 1995 |
| [S209] | anti-TNF monoclonal antibody | No benefit | van Hensbroek *et al.,1996* | 1996 |
| [S210] | Dichloroacetate | No benefit | Krishna *et al.,* 1996 | 1996 |
| [S211] | Pentoxyfylline | No benefit | Hemmer *et al.,* 1997 | 1997 |
| [S212] | Pentoxyfylline | No benefit | Looareesuwan *et al.,* 1998 | 1998 |
| [S213] | Deferoxamine | No benefit | Thuma *et al.,* 1998 | 1998 |
| [S214] | High dose phenobarbital | Harmful | Crawley *et al.,* 2000 | 2000 |
| [S215] | Deferiprone | No benefit | Mohanty *et al.*, 2002 | 2002 |
| [S216] | N-Acetylcysteine | beneficial | Watt *et al.,* 2002 | 2002 |
| [S217] | Pentoxyfylline | No benefit | Das *et al.,* 2003 | 2003 |
| [S218] | N-Acetylcysteine | No benefit | Treeprasertuk *et al.,* 2003 | 2003 |
| [S219] | Dichloroacetate | Reduced blood lactate | Agbenyega *et al.,* 2003 | 2003 |
| [S220] | Curdlan sulphate | No benefit | Havlik *et al,* 2005 | 2005 |
| [S221] | Albumin | No significant benefit | Maitland et al.,2005 | 2005 |
| [S222] | Gelofusine | No significant benefit | Akech *et al.,* 2006 | 2006 |
| [S223] | Mannitol | No benefit | Namutangula *et al.,* 2007 | 2007 |
| [S224] | N-Acetylcysteine | No benefit | Charunwatthana *et al.,* 2009 | 2009 |
| [S225] | Erythropoetin | No benefit | Picot et al., 2009 | 2009 |
| [S226] | Pentoxyfylline | Harmful | Lell *et al.,* 2005 | 2010 |
| [S227] | Activated charcoal | No benefit | De Souza et al., 2010 | 2010 |
| [S228] | Mannitol | Harmful | Mohanty *et al.,* 2011 | 2011 |
| [S229] | Bolus high volume albumin/saline | Harmful | Maitland *et al.,* 2011 | 2011 |
| [S230] | Early enteral feeding | Harmful | Maude et al., 2011 | 2011 |
| [S231] | Vitamin A | No benefit | Mwanga-Amumpaire *et al*.,2012 | 2012 |
| [S232] | Levamisole | No benefit | Maude *et al.,* 2014 | 2014 |
| [S233] | Nitric Oxide | No benefit | Hawkes *et al.,* 2015 | 2015 |
| [S234] | Nitric Oxide | No benefit | Mwanga-Amumpaire *et al., 2015* | 2015 |
| [S235] | Delayed iron therapy | No benefit | Cusick *et al.,* 2016 | 2016 |
| [S236] | Delayed iron therapy | No benefit | Cusick *et al.,* 2020 | 2020 |
| [S237] | Delayed iron therapy | No benefit | Ssemata *et al.,* 2020 | 2020 |
| [S238] | Aggressive antipyretic therapy | Concerns over harm | Birbeck et al., 2024 | 2024 |
